# Chemistry, Processing, Properties, and Applications of Rubber Foams

**DOI:** 10.3390/polym13101565

**Published:** 2021-05-13

**Authors:** Ehsan Rostami-Tapeh-Esmaeil, Ali Vahidifar, Elnaz Esmizadeh, Denis Rodrigue

**Affiliations:** 1 Department of Chemical Engineering, Université Laval, Quebec, QC G1V 0A6, Canada; ehsan.rostamitapehesmaeil.1@ulaval.ca; 2 Department of Polymer Science and Engineering, University of Bonab, Bonab 5551761167, Iran; elnaz.esmizadeh@uwaterloo.ca

**Keywords:** rubber, foam, morphology, curing, characterization, applications

## Abstract

With the ever-increasing development in science and technology, as well as social awareness, more requirements are imposed on the production and property of all materials, especially polymeric foams. In particular, rubber foams, compared to thermoplastic foams in general, have higher flexibility, resistance to abrasion, energy absorption capabilities, strength-to-weight ratio and tensile strength leading to their widespread use in several applications such as thermal insulation, energy absorption, pressure sensors, absorbents, etc. To control the rubber foams microstructure leading to excellent physical and mechanical properties, two types of parameters play important roles. The first category is related to formulation including the rubber (type and grade), as well as the type and content of accelerators, fillers, and foaming agents. The second category is associated to processing parameters such as the processing method (injection, extrusion, compression, etc.), as well as different conditions related to foaming (temperature, pressure and number of stage) and curing (temperature, time and precuring time). This review presents the different parameters involved and discusses their effect on the morphological, physical, and mechanical properties of rubber foams. Although several studies have been published on rubber foams, very few papers reviewed the subject and compared the results available. In this review, the most recent works on rubber foams have been collected to provide a general overview on different types of rubber foams from their preparation to their final application. Detailed information on formulation, curing and foaming chemistry, production methods, morphology, properties, and applications is presented and discussed.

## 1. Introduction

Cellular materials, commonly known as foams, both are widely used by man and appear in nature to reduce the weight of materials while increasing their strength [1]. Typical examples taken from nature are wood, cork, plant stalk, cancellous bone, sponge, coral, etc. [2], so the production of synthetic cellular materials was devoted to mimic nature leading to the development of one of the most important and widely used man-made class of materials with a wide range of applications in automobiles, aeronautics, packaging, medicine, construction, etc. [3,4,5,6,7,8,9]. 

Foams or sponges are generally defined as composite materials made from gaseous voids (dispersed phase) surrounded by a denser matrix (continuous phase) which is usually a liquid or a solid [10,11]. Depending on the matrix type, these foams can be classified into metal, concrete, biobased, polymeric, etc. [12,13,14,15,16,17]. Recently, polymeric foams have drawn a great deal of interest because of their good strength, high surface area, low weight and excellent energy damping properties performance, as well as good electrical, thermal and acoustic insulation combined with impact mitigation and cost reduction compared to other conventional foams [18,19]. In addition, it is easier to control their structure and properties [20].

The first cellular polymer reaching commercial markets was a rubber sponge developed as early as 1914 [21]. It was made by the addition of gas generating chemicals, like sodium and ammonium carbonate or sodium polysulfide, to natural rubber (NR) latex. However, the synthesis of thermoplastic foams started with polystyrene (PS) in 1931 [21]. Besides PS, other important thermoplastic foams, like polyethylene (PE), polypropylene (PP), polyvinyl chloride (PVC), are used for different applications depending on their properties [22,23,24,25]. The development of polymeric foams is still ongoing, but some characteristics of rubber foams are making them preferable. For example, rubber foams have higher flexibility, resistance to abrasion and deformability (especially at low temperature) making them key materials in our everyday life. As technological development is made, their uses will continue to increase to a wider range of conditions such as high/low temperatures and pressures, vibration frequency, radiation or chemical environments [26,27,28]. In other words, their great elasticity, strength-to-weight ratio, impact and energy absorbing capabilities have secured their widespread use in several applications based on their chemical, mechanical, physical and thermal properties [29,30]. 

Based on the large body of literature now available on rubber foams, the main objective of this review is to provide a general overview on the relation between rubber formulation and foaming conditions. In particular, the curing kinetics, foaming chemistry and processing conditions are related to the final foam’s morphology, while their general properties and applications are discussed to get a state of the current knowledge and openings for future development.

## 2. Structural Features

### 2.1. Open and Closed Cell Structures

Rubber foaming relies on the expansion of a gaseous phase dispersed throughout the rubber melt. In porous materials, the pores are usually divided into two types: open cells which are interconnected throughout the material and also connected to the ambient environment, and closed cells where the gas is dispersed as separate/individual bubbles throughout the matrix and are isolated from the outside [31]. According to the reported qualitative findings, open cell foams are predominant from low-viscosity materials, while a closed cell structure is mostly seen for highly viscous conditions [32]. According to their structural differences, their properties as well as their applications are varied. Open cell foams usually have higher porosity, lower weight and cost which are used as supports, fuel tank inserts and loudspeakers. Due to these interconnections between the cells, they have the ability to allow fluids pass through their structure, which is useful for membranes or filters in separation processes [33]. On the other hand, closed cell foams, which are denser and more expensive, show higher mechanical strength and lower moisture absorption which can be used as thermal or electrical insulators. Closed or open cell structures can be prepared using a chemical (CFA) and/or physical (PFA) foaming agent. 

**Figure 1:**
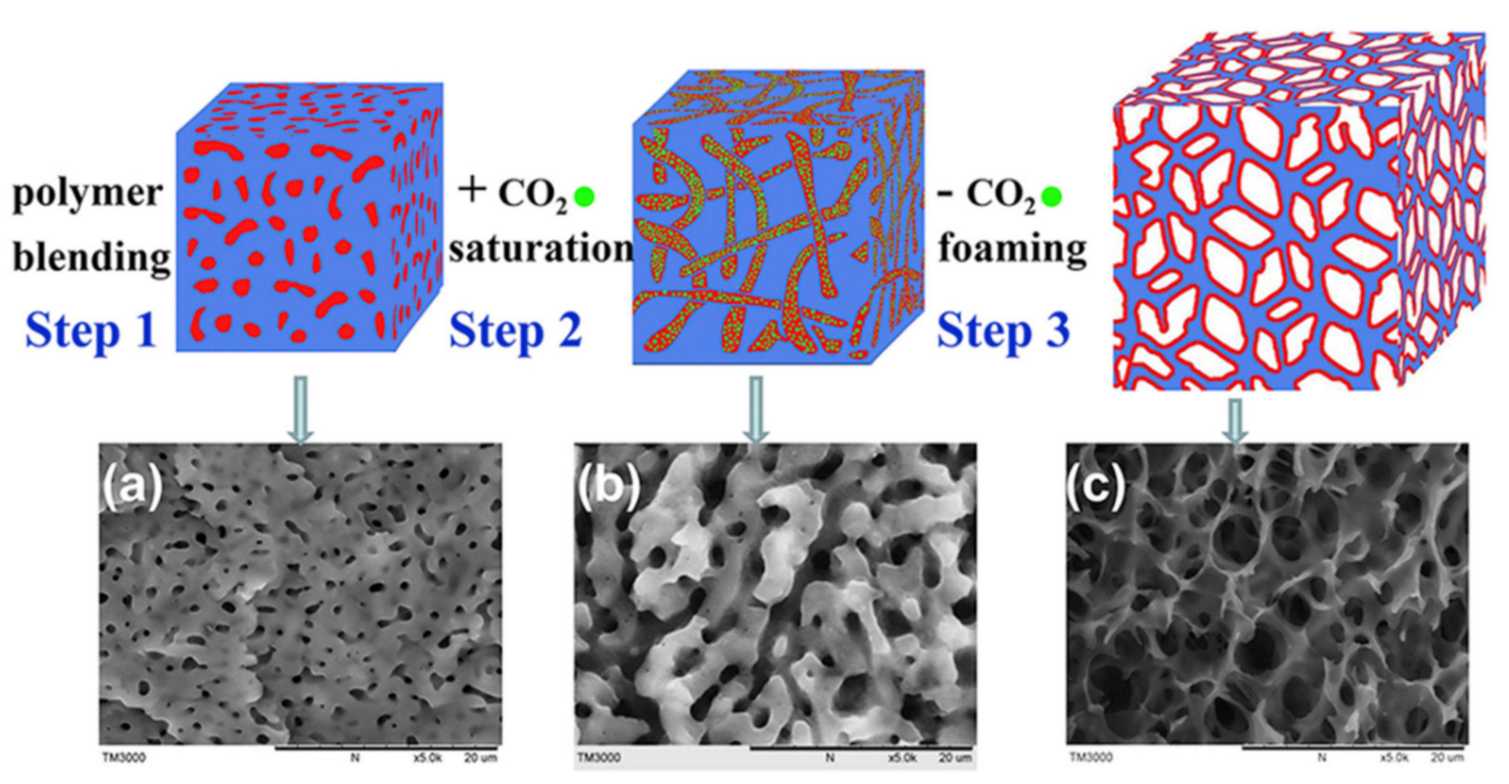
Schematic representation and SEM images of the steps leading to an open cell PP/EPDM foam [34].

Kong et al. prepared an open cell PP/EPDM foam using carbon dioxide (CO_2_) as a physical foaming agent [34]. They found that using a polymer blend with a co-continuous morphology, for which one phase is CO_2_-philic and has a low glass transitions temperature or melting point, can lead to the production of an open cell structure using CO_2_ (Figure 1). In another work using CO_2_ as a PBA, the formation of a closed-cell structure was possible using a poly(ethylene-*co*-octene) rubber as the matrix [35]. Ethylene vinyl acetate (EVA) foams based on supercritical carbon dioxide (scCO_2_) using various sorption temperatures showed that the rapid expansion of EVA below and above its melting point led to open cell and collapsed closed cell morphologies, respectively [36]. Inorganic CFA, like ammonium carbonate and sodium bicarbonate, mainly release CO_2_ [37]. Their endothermic decomposition produces a slow gas generation producing an open cell structure. On the contrary, organic CFA, which are mostly exothermic, mainly produce closed cell morphologies [35,38].

Another important additive in rubber foams is incorporation of nanofillers which can act as nucleating agents to control the foam porosity [39]. Shojaei et al. prepared ethylene propylene diene monomer (EPDM) foams with 4,4′-oxybis(benzene sulfonyl hydrazide) (OBSH) and observed that nanoparticles addition can lead to different close/open cell ratio depending on their geometry [40]. For instance, carbon nanotubes (CNT), with their tubular structure, produced closed cells at low content, but higher open cell content was observed with increasing concentration. Conversely, organo-modified nanoclay (NC), having a plate-like shape, hindered the formation of open cells and their number decreased with increasing NC content. Finally, the spherical shape of nanosilica did not have a significant effect on open/closed cell structure of the foams.

### 2.2. Cell Size, Cell Density and Cell Wall Thickness

Controlling the cell size, density and uniformity of rubber foams is a challenging issue because the structure has a direct effect on the properties of rubber foams. Progress in the field of foam processing technology led to the fabrication of foams with different cell sizes including macrocellular foams (cell size > 100 µm), microcellular foams (cell size ≤ 100 µm and cell density > 10^9^ cells/cm^3^) and nanocellular foams (cell size < 1 µm and cell density >10^15^ cells/cm^3^). It has been shown that foams with smaller and more uniform cell size offered improved properties, especially mechanical ones [41]. For a quantitative analysis of the foam morphology, several parameters like *D_n_*, *D_w_*, *PDI* and *ρ_cell_* can be used as [42]:

(1) $$D_{n}=\frac{\sum \left(n_{i}\;D_{i}\right)}{\sum n_{i}}$$

(2) $$D_{w}=\frac{\sum \left(n_{i}\;D_{i}^{2}\right)}{\sum (n_{i}\;D_{i})}$$

(3) $$PDI=\frac{D_{w}}{D_{n}}$$

(4) $$\rho_{cell}=\frac{\rho_{bulk}}{\rho_{foam}}\;{\left(\frac{\sum n_{i}}{A}\right)}^{\frac{3}{2}}$$

where *n_i_* is the number of cells with a diameter *D_i_*, *ρ_cell_* is the cell density (number of cell per unit volume), *A* is the surface of the foam, *ρ_bulk_* is the density of the unfoamed matrix and *ρ_foam_* is the foam density. These data are usually obtained from the cell structure using 2D scanning electron microscopy (SEM) images.

In order to control the foams’ cell size and cell density, nanoparticles can be added as nucleation agents [43]. In general, nanoparticles addition results in lower cell size and cell wall thickness with higher cell density [44,45]. In this case, the nanoparticles affect both the nucleation and growth steps. On the one hand, nanoparticles act as nucleation agents and facilitate the formation of gas cells via heterogeneous nucleation. On the other hand, the addition of nanoparticles increases the viscosity limiting bubble growth due to higher matrix viscosity. During the foaming process, the pressure from the released gas (foaming agent) is the driving force for cell growth [46,47]. This is combined with lower average cell size and cell wall thickness with narrower cell size distribution while the number of cells per unit volume increase. The amount and distribution of the nano-reinforcements are also important factors to determine the foam quality. The influence of nanoparticle content is explained in detail in Section 3.4, but a nonuniform distribution of nanoparticles leads to a nonuniform cell size distribution. Another factor controlling the cell structure is the content of chemical and physical foaming agent [48,49]. As the concentration of foaming agent increases, the cells become smaller, finer, and more uniform. This is especially the case for CFA since their decomposition generates more gas and nucleation points [50]. Consequently, the cell density increases resulting in a smaller average cell size since the total amount of gas is constant. On the other hand, the cell size and cell density are mainly determined by the diffusivity and solubility of PFA. Yan and coworkers reported that when the saturation pressure increases (fixed temperature), the cell size decreases resulting in narrower cell size distributions [51]. Hence, increasing the CO_2_ saturation pressure in their case led to more molecules being absorbed by the silicone rubber matrix leading to a more important homogeneous nucleation effect producing a higher number of larger cells.

## 3. Rubber Foaming Formulation

Several parameters must be controlled to produce a foams with a specific morphology and properties [52]. These parameters are divided in two categories. The first category includes parameters related to formulation (type and content) including the rubbers, accelerators, fillers, and foaming agents. The second category is associated to processing conditions (batch or continuous) like temperature, pressure, time, number of steps and their sequence [53,54,55]. All these parameters can control the foam microstructure leading to different physical and mechanical properties of the final product. It is very important to choose an optimum formulation-processing combination to achieve suitable foams properties. The formulation parameters are presented next.

### 3.1. Type and Amount of Foaming Agents

Commercial and industrial applications require rubber foams with appropriate physical and mechanical properties, as well as uniform cell structures. An accurate control of the foam structure directly depends on the proper selection of foaming and curing agents to achieve a balance between gas production and degree of curing [56]. Two types of foaming agents (physical and chemical) can be used for rubber foam production, each having pros and cons. In general, physical foaming agents (PFA) are inexpensive and do not leave any solid residues which might affect the color or properties of the polymer. On the other hand, some have safety issues (explosion and flammability) and environmental issues (ozone depletion potential, greenhouse emission), as well as working with gases requires specific additional equipment (high pressure pump and flow meter) and thus more complex processes are required. It is also more complex to separate the foaming and crosslinking steps make it more difficult to control the foaming [57]. These conditions increase the interest of using chemical foaming agents (CFA) which are mainly powders. Despite their wide use, easier handling, and manufacturing (dosing), several CFA pose health hazards. In addition, unwanted byproducts and high cost have restricted their use. However, despite all these problems, CFA are more commonly used in the rubber foam industry.

#### 3.1.1. Physical Foaming Agent (PFA)

**Scheme 1:**
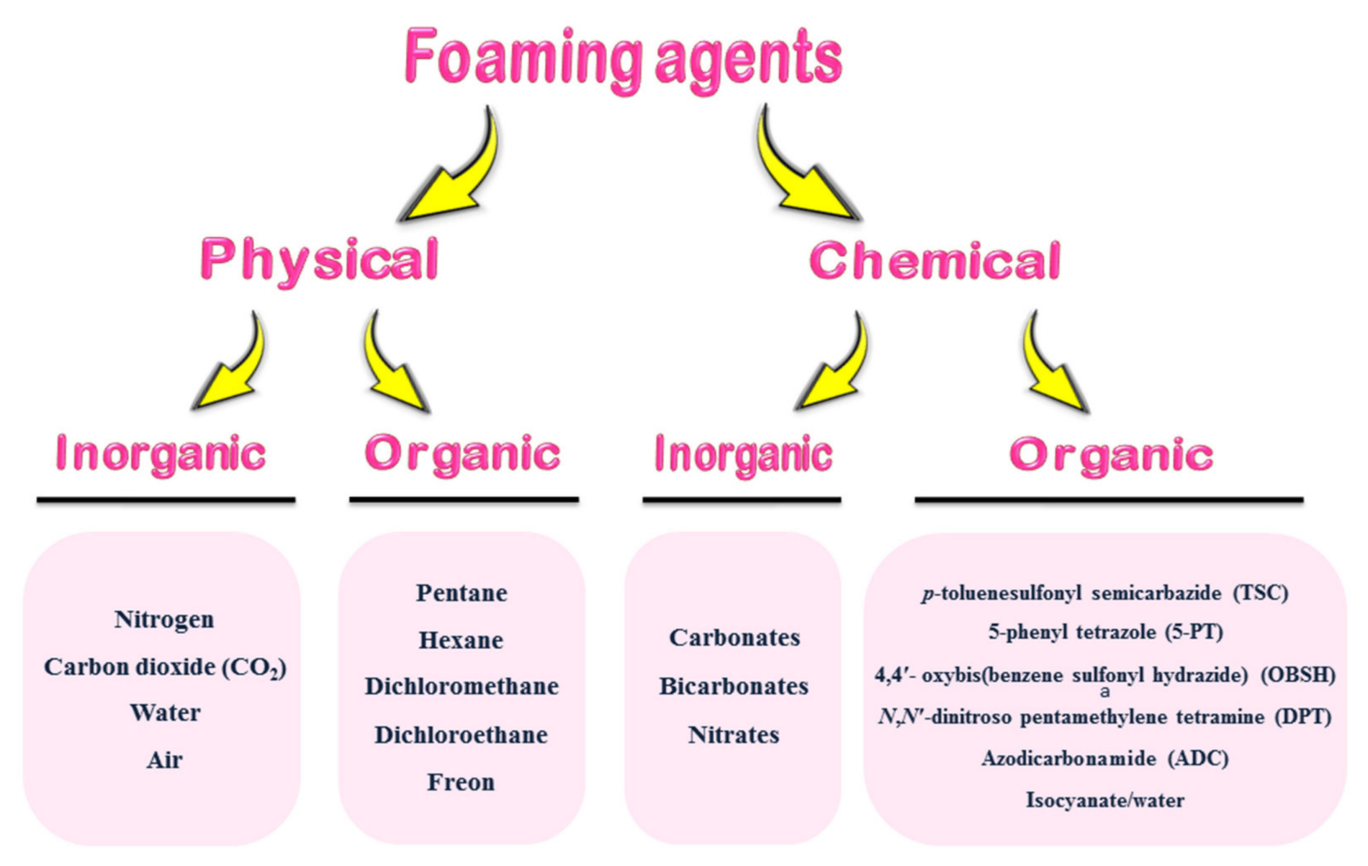
Classification of foaming agents.

Some volatile liquids are able to generate gases by altering their physical state (liquid to gas) to produce a foam [58]. On the other hand, some compressed gas can be injected in the matrix under high pressure [59]. A good physical foaming agent should be nontoxic, nonflammable, odorless and noncorrosive with high vapor pressure and solubility. They must also be thermally stable and chemically inert and must not affect the physical or chemical properties of the rubber matrix. PFA are classified into inorganic and organic molecules (Scheme 1). The most important inorganic PFA are nitrogen (N_2_), CO_2_, water and air [60,61,62,63,64,65], while organic PFA are based on pentane, hexane, dichloromethane, dichloroethane, freon and similar structures [66,67].

According to the literature, several substances, such as compressed inert gases and supercritical fluids, in particular supercritical carbon dioxide (scCO_2_), have been proposed as inorganic PFA [68]. Compressed gases are incorporated into the matrix by using special extruders and screws for gas injection [69]. The foaming process can be decomposed into four steps: gas dissolution, bubble nucleation, bubble growth and structure stabilization. The latter in rubber foams can be achieved by vulcanization (curing). Although CO_2_ and N_2_ are mainly used, scCO_2_ has several advantages including a tunable solvent strength, high diffusion rates and good solubility due to its low critical temperature (*T*_c_ = 304.15 K) and pressure (*P*_c_ = 7.38 MPa) [70,71,72]. N_2_ is also commercially highly used because of its inertness and high availability with low cost. 

Jacobs et al. produced EVA foams with scCO_2_ and found that increasing the sorption pressure led to denser foams with smaller cell sizes after rapid expansion [36]. Tessanan and coworkers used scCO_2_ to prepare natural rubber (NR) foams [73]. They reported that increasing the scCO_2_ saturation time and pressure resulted in lower average cell size and volume expansion ratio, leading to significantly smaller cell size and narrower distribution.

Until some years ago, chlorofluorocarbon (CFC) and hydrochlorofluorocarbon (HCFC) were the main organic PFA for the foam industry, but the main concern about them was the presence of chlorine in their structure, which was known to attack the ozone layer. Furthermore, they have very high global warming potential (GWP). Therefore, they were phased out and the industries turned to hydrofluorocarbon (HFC). However, HFC are strong greenhouse gases and will no longer be used as replacements. Hydrofluoroolefin (HFO) were recently proposed since they do not have ozone depletion potential, but need more time before any conclusion about their long-term environmental effects is understood [74].

#### 3.1.2. Chemical Foaming Agent (CFA)

CFA are widely used to produce elastomer foams. They are also classified as inorganic and organic types (Scheme 1) [38]. Inorganic CFA, mostly leading to the formation of open cells, are based on ammonium, sodium and potassium carbonates, bicarbonates and nitrates [75,76,77,78,79]. They usually release CO_2_ and water under the action of heat (thermal decomposition) and acids (accelerators). Most of the aforementioned inorganic CFA decompose endothermically; i.e., they absorb energy to react. These reactions are usually slow leading to the formation of open cell foam with a coarse cell structure [80]. To produce a finer cell structure, bicarbonates are combined with an acid activator like hydrogen phosphates, tartaric acid or citric acid [81]. A typical dosing is 5 to 10 phr (parts per hundred rubber).

**Figure 2:**
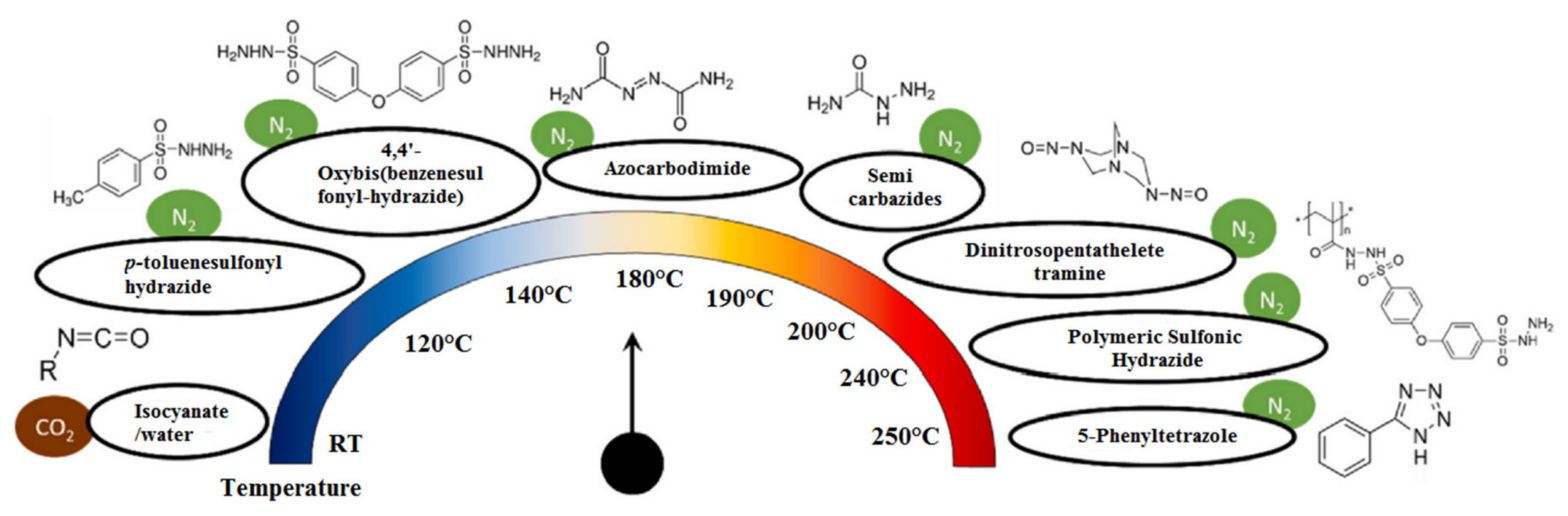
Typical organic CFA and their gas produced depending on the temperature [85].

On the other hand, organic CFA mainly react exothermically, i.e., they release energy while decomposing. The main organic CFA are *p*-toluenesulfonyl semicarbazide (TSSC), 5-phenyl tetrazole (5-PT), OBSH, *N*,*N*′-dinitroso pentamethylene tetramine (DPT), azodicarbonamide (ADC) and isocyanate/water which release a gas mixture of N_2_, CO_2_, carbon monoxide (CO), ammonia and water. However, the main CFA is ADC and the predominant gas released is N_2_ [82,83,84]. Figure 2 presents a list of typical organic CFA and their gas produced depending on the temperature. The information is mainly based on the work of Coste and coworkers [85].

For CFA, the molecules breakdown via a series of reactions after reaching the decomposing temperature and the heat released can activate adjacent particles. This cascade-like process (autocatalytic reaction) leads to a fast foaming process mainly resulting in closed cell foams with uniform cell structures [86]. ADC is a versatile and well-known organic CFA because of the ability to tailor its decomposition temperature (pure ADC’s decomposition temperature is around 210 °C) and high gas yield efficiency (231 cm^3^ of gas generated per each gram of ADC) [87,88]. Although time and temperature have a direct effect on the thermal decomposition behavior, particle size, kind and amount of activator, and the quality of distribution of the activator and CFA also affect the decomposition temperature [89]. By reaching its decomposition temperature, ADC starts to generate gas molecules to expand the rubber matrix [90].

**Figure 3:**
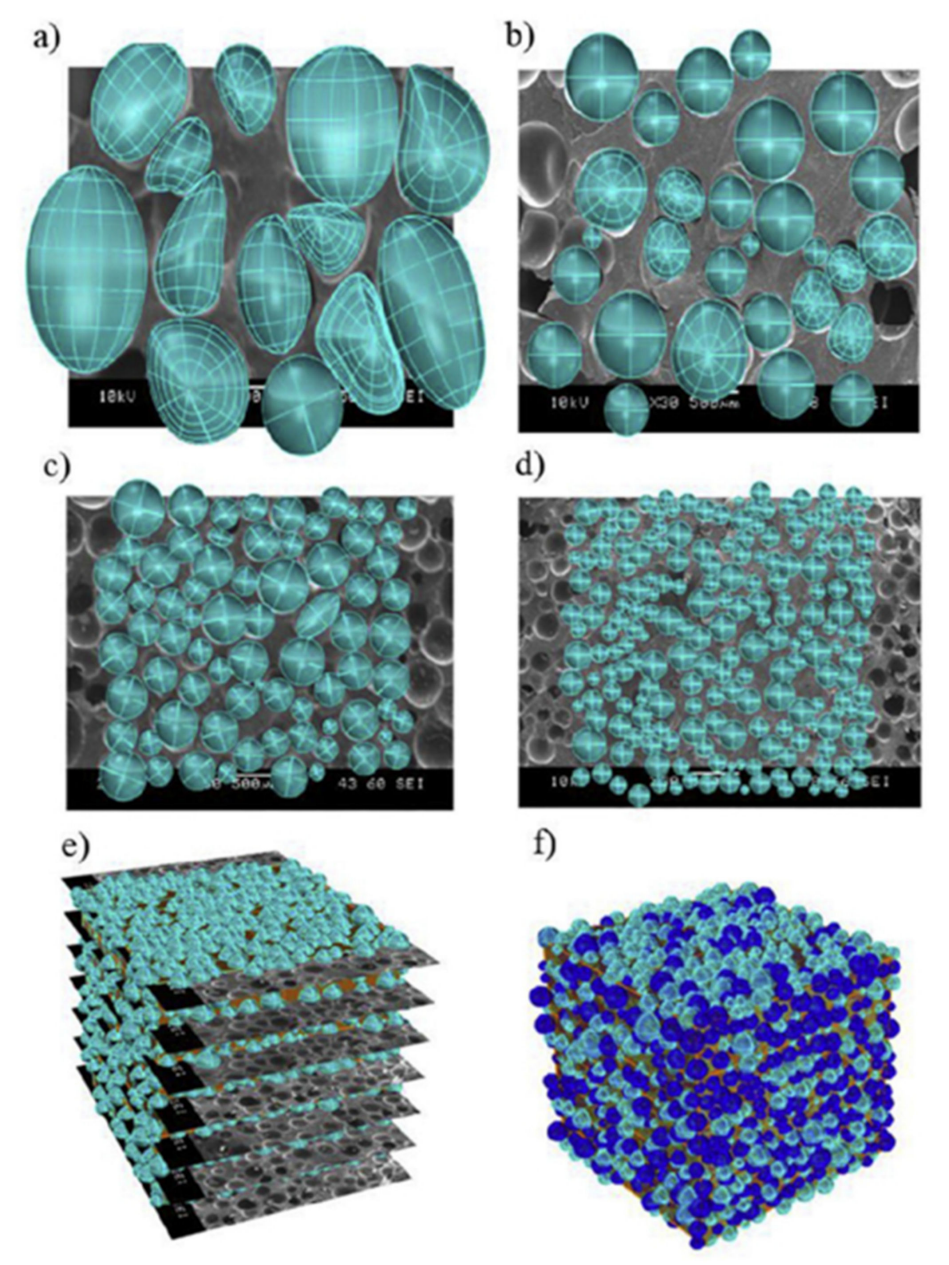
Real 2D geometry models from SEM images for NR foams with different foam relative density of: (**a**) 0.3, (**b**) 0.4, (**c**) 0.45, (**d**) 0.5, as well as (**e**) the real 3D geometry obtained from 2D data for a sample with a relative density of 0.5 and (**f**) the final 3D structure for a foam with a relative density of 0.5 [92].

All the foaming agents can be used alone or together. For example, Hongling and coworkers found that using a mixture of ADC/OBSH was more effective than ADC alone because chloroprene rubber (CR) foams with higher porosity, larger cell size and more uniform cell distribution were produced [90]. Moreover, the foams prepared with ADC/OBSH had better tensile strength and tear strength than neat ADC, as well as higher hardness. Charoeythornkhajhornchai et al. reported that increasing ADC content from 3 to 4 phr in NR foams led to smaller cell size because of faster vulcanization rate (less time for cell growth) owing to the presence of amine species (curing accelerators) as ADC decomposition by-products [91]. However, further ADC increase (5 and 6 phr) produced cell coalescence because of a high gas content and internal cell pressure. They also stated that 4 phr ADC produced the lowest thermal expansion coefficient due to better cell uniformity and smaller cell size (low coalescence). Heydari et al. generated closed cell NR foams by ADC and use their data to validate various numerical models [92]. They achieved relative foam densities between 0.3 and 0.5 by enhancing the mold filling ratio (compound weight inside a fixed mold volume). Their SEM analysis (Figure 3) revealed that increasing the filling ratio resulted in lower average cell sizes (from 530 to 234 μm), narrower cell size distributions (from 225–800 to 50–400 μm), and higher cell densities (from 29 to 294 cell/mm^3^). In particular, they simulated their 3D foam structures using real 2D SEM images to predict the mechanical behavior of NR foams via finite element methods (FEM). Their FEM outcomes at the macroscopic level were in good agreement with mechanical test results for the range of conditions tested.

### 3.2. Type of Rubbers

The curing characteristics and cellular structure, as well as the mechanical and physical properties of the foams, can be controlled by a careful selection of the rubber type. So far, various rubber foams were reported based on NR, EPDM, polyisoprene rubber (IR), CR, styrene butadiene rubber (SBR), EVA, isobutylene rubber (IIR), acrylonitrile butadiene rubber (NBR), polyurethane (PU), chlorinated polyethylene rubber (CPE), silicone rubber (SR), etc. [93,94,95,96,97,98]. A summary of the main rubber types with their properties and molecular structure is presented in Table 1.

**Figure 4:**
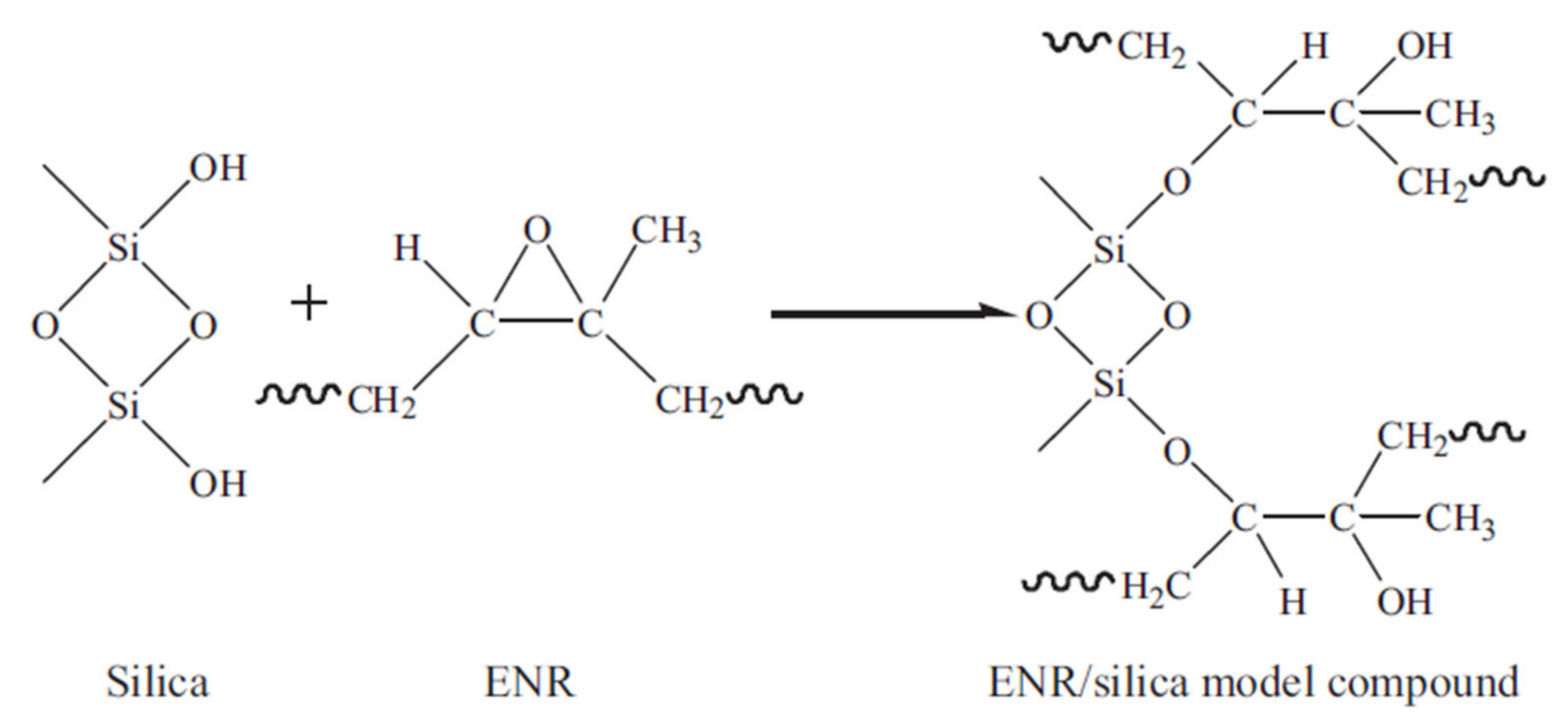
The ring-opening reaction between an epoxy group of ENR and Si-OH on the surface of silica [107].

Among the rubbers, NR is the main matrix for foam production due to its natural origin (biosourced), availability, renewability and appropriate mechanical and electrical properties [99]. Good mechanical properties include high elastic properties, high resilience and damping behavior, high tensile strength, low compression set, resistance to tear and abrasion, but poor chemical resistance and processing ability [50,100]. NR has four possible microstructures in its molecular chains: cis-1,4-, trans-1,4-, 1,2- and 3,4-polyisoprene [101], but the main component is cis-1,4-polyisoprene. NR is obtained in a latex from trees like *Hevea brasiliensis* (Para rubber trees) and banyan fig trees (*Ficus bengalensis*) or various plants like guayule shrub (*Parthenium argentatum Gray*) and the Russian dandelion (*Taraxacum koksaghyz*), but contains various impurities (mainly proteins, amino acids, phospholipids and gel components) [102]. The standard Malaysian rubber (SMR) categorized NR latex into various grades according to the amount of main impurities (dirt, ash, nitrogen, volatile matter). Nitrogenous materials in the rubber are related to proteins, as determined through its nitrogen content, can provide an estimation of the protein content in the rubber [103]. SMR-L, SMR-5, SMR-10 and SMR-20 are the main NR grades and the main impurities are listed in Table 2. Another important grade is epoxidized natural rubber (ENR) which is a chemically-modified grade of NR obtained via epoxidation [104]. The presence of epoxy groups on the backbone increases the NR polarity leading to faster curing rate and better final properties of the foams. Ariff et al. investigated the effect of various NR grades on the morphological, mechanical and physical properties of NR foams [105]. They used three different NR grades (SMR-L, SMR-10 and ENR-25: ENR with 25 mole% epoxide). Their results showed that both unmodified grades (SMRL and SMR-10) did not exhibit significant difference in cell size, crosslink density (CLD), rate of expansion, rate of curing and mechanical properties because of their similar chemical structure [106]. On the other hand, ENR-25 foams had smaller cell size, higher cell wall thickness, higher density, and better mechanical properties. This behavior was attributed the epoxide groups on the ENR backbone reacting with the carbon double bond sites and increasing the CLD. Xu et al. found that the mechanical properties of NR/silica were improved after ENR addition [107]. This improvement was attributed to the ring-opening reaction between the epoxy groups of ENR and the Si-OH groups on the silica surface which improved the dispersion of silica in the rubber matrix and enhanced the interfacial interactions between rubber and silica (Figure 4). Salmazo et al. produced SMR-L and ENR-25 foams separately in the presence of ADC and cured with different electron beam irradiation doses (50, 100 and 150 kGy) [108]. Their results revealed that ENR foams had higher cell nucleation rates and less cell degradation than those produced from SMR-L alone. This was attributed to the presence of epoxide groups in the cured ENR foam promoting a higher degree of curing. Consequently, the ENR foams had more uniform porous structures and smaller cell sizes.

SBR is a synthetic rubber derived from petroleum. It was initially developed as an alternative for NR. SBR is fabricated by the copolymerization of ~75% butadiene (CH_2_=CH-CH=CH_2_) and 25% styrene (CH_2_=CHC_6_H_5_) [110]. Some SBR properties, like excellent compression set, enhanced crack resistance, abrasion-resistance, wear resistance and cost reduction, enable its industrial use due to improved aging properties and thermal insulation [111]. However, SBR foams have rarely been reported because of their very high viscosity and shrinkage [112]. The effect of CFA type (OBSH and ADC) and content (0, 2, 4, 6, 8 and 10 phr) on the curing characteristics, mechanical and morphological properties of cellular NR/SBR was studied by Wimolmala et al. [113]. They reported that 4 phr was the optimum concentration of foaming agent (for both OBSH and ADC), but OBSH led to higher curing rate (lower curing time) compared to ADC. Furthermore, increasing the foaming agent content led to lower elastic recovery and higher resilience of NR/SBR foams. Shao et al. were able to decrease the shrinkage of SBR foams to 2.25% by using sulfur and dicumyl peroxide (DCP) producing a double crosslinking system [96]. Their study reported that shrinkage mainly depended on a synergy effect between both cross-linkers. SBR/RR (recycled rubber) foams with different ratios (100:0, 80:20 and 60:40) were prepared with sodium bicarbonate by Algaily and coworkers [114]. 

Increasing the RR concentration resulted in better mechanical properties (higher elongation at break and tensile strength), higher curing characteristics (maximum/minimum torque and crosslink density), and higher foam density. Changing the RR:SBR ratio resulted in a shift of the resonant frequency from 500 Hz (100:0) to a higher frequency of 800 Hz (80:20 and 60:40). Bahadar et al. prepared a single wall carbon nanotube (SWCNT)-reinforced EPDM/SBR blend for shock absorbing applications [115]. The introduction of SWCNT in an EPDM/SBR matrix led to a substantial improvement of the storage modulus (80%), but a 27% decrease in the loss modulus for the highest SWCNT content tested (0.6 mass%). Rheological studies showed that adding SWCNT into a rubber blend increased the loss factor (tan δ = E′/E″) but decreased the mixing torque. Degradation with increasing filler/matrix ratio was observed in compressive strength and energy absorption efficiency. All these effects were related to the superior mechanical strength, more uniform dispersion, and long-lasting bonding between the matrix and SWCNT.

EPDM is an unsaturated polyolefin rubber obtained by the copolymerization of ethylene, propylene and a nonconjugated diene (ethylidene norbornene, dicyclopentadiene or 1,4 hexadiene) which provides crosslinking sites for vulcanization [116]. The presence of propylene in the EPDM backbone prevents the formation of crystallinity to keep a higher amorphous content. The lack of unsaturated double bond in the main-chain of EPDM provides great heat aging resistance, chemical stability, ozone, UV and oxidation resistance, as well as high load capacity and high resistance to break down during [116,117]. EPDM has attracted high attention for outdoor applications such as automotive sealing systems, wire materials, building profiles, white sidewalls of tires, electric-electronic components, roofing sheets and sporting goods. The selection of an appropriate vulcanization systems in combination with a suitable foaming agent has always been a challenge for EPDM foams because of its slow vulcanization rate [118]. By blending EPDM with NR, Lewis and coworkers found that the structure, properties and cure characteristics of EPDM/NR foams are affected by the amount of NR in the blend [119]. The scorch time and cure time decreased with increasing NR content due to the high reactivity of the double bonds and methyl group enhancing the activity of the double bond in NR [120]. EPDM/halloysite nanotube (HNT) nanocomposite foams were produced using a batch process in an autoclave with scCO_2_ as PFA by Lee and coworkers [121]. They achieved microcellular foams with an average cell size of 7.8 µm and a cell density of 1.5 × 10^10^ cell/cm^3^, revealing that HNT acted as an effective nucleating agent for the foaming process. This microcellular elastomeric nanocomposite foam could potentially be used in a variety of industrial applications involving gaskets and seals for automotive and electrical enclosures. Suntako investigated the effect of synthesized ZnO nanoparticles by a precipitation method as sulfur vulcanization activator compared with conventional ZnO on the curing characteristics and morphology of EPDM foams [122]. It was shown that EPDM foams based on synthesized ZnO nanoparticles exhibited higher ultimate torque, compressive load and hardness with increasing nanoparticles content, while the optimum cure time and scorch time decreased. The cell structure of the EPDM foams was more spherical, and the cell size decreased with increasing synthesized ZnO nanoparticles. It was also possible to decrease the amount of synthesized ZnO from 5 to 3 phr (40%) because the ZnO nanoparticles have much higher specific surface areas. Ma et al. developed EPDM composite foams with various SR ratios to determine its effect on the wear resistance, structure, curing, rheological and mechanical properties of the blends [27]. The torque values and curing level were both increased with SR concentration. The mechanical properties of the composite foams were all improved with SR addition, except for the elongation at break. More importantly, the wear resistance and compression set were substantially enhanced with increasing SR content. Bio-EPDM foams with various ratio of tungsten bronze nanorod (TBNR) were fabricated to improve the thermal insulation properties for application in highly functional eco-friendly diving wetsuits [123]. The crosslinking delay and stabilization of the foaming speed upon TBNR addition controlled the bubble growth and induced the formation of smaller cells with a more uniform size distribution. The processing stability of the foam was also improved leading to an elastomer foam with excellent thermal insulation, flexibility, elastic properties, and foam stability without significant changes in mechanical properties. Moreover, an excellent photothermal stability after light irradiation indicated that the material developed was suitable for thermo-functional wetsuits in water sports and activities.

EVA foams have been industrially applied in a broad range of products like shoe soles and midsoles, sports equipment, insulation materials and drug delivery systems because they are highly durable, very comfortable and soft [36]. However, some disadvantages, such as high density and low physical properties, restricted further applications [28]. To overcome these disadvantages, Park et al. blended EVA with ethylene-1-butene copolymer (EtBC) to improve the physical properties of EVA foams including tensile strength, rebound resilience and compression set [124]. Kim et al. achieved low density, high rebound resilience and tear strength of EVA foams (at optimal crosslinking temperature) through blending with NR [125].

The random substitution of chlorine atoms on the PE backbone results in its transformation from a thermoplastic material to a rubber material (CPE) by suppressing crystallization. CPE has high resistance to hydrocarbon oil, heat and weathering from the addition of chlorine atoms on the PE backbone [126]. Moreover, the presence of both nonpolar groups (unmodified methylene units) and polar groups (chlorinated methylene *co*-units) in the CPE’s backbone increase its compatibility to blend with either polar or nonpolar polymers for a specific sets of properties and cost advantages purposes [127]. Zhang and coworkers prepared a series of foams to investigate the effect of CPE/EVA ratio on the curing, foaming and mechanical properties [128]. They found that increasing the EVA content had a negligible effect on the scorch and curing time, but higher hardness with lower rebound resilience and shrinkage ratio were obtained. The expansion ratio and void fraction increased with increasing EVA content, while the cell density decreased from 100:0 to 50:50 of CPE:EVA, and then considerably increased from 70:30 to 90:10. 

**Figure 5:**
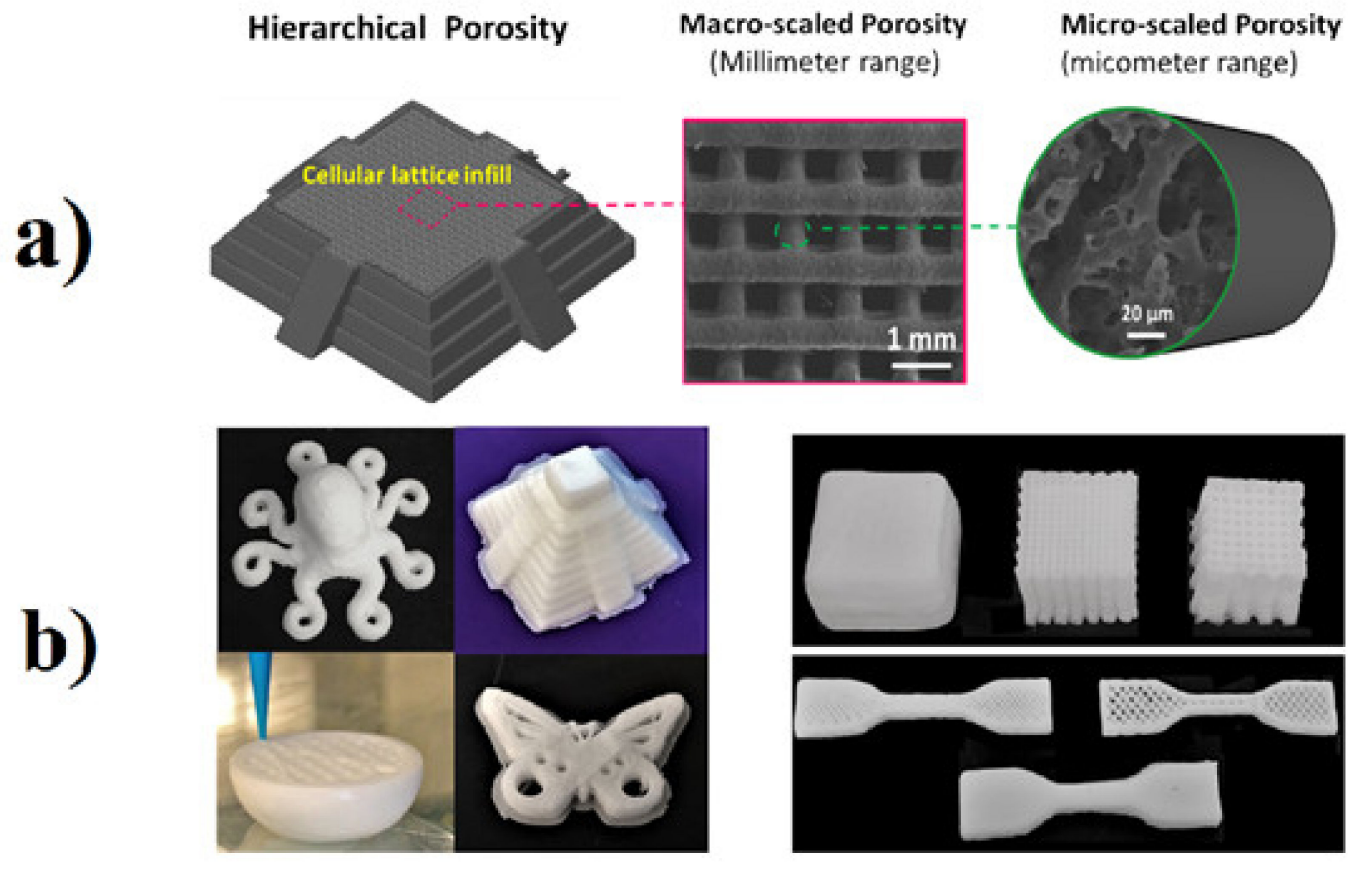
(**a**) Illustration of hierarchical porosity in 3D printed objects. Left: schematic drawing. Middle: macro-pores under SEM. Right: micro-pores under SEM. (**b**) Optical images of 3D printed PDMS sponge structures [140].

Silicone (polyorganosiloxane) consists of alternating silicon and oxygen atoms (siloxane units) with hydrocarbon side radicals combined directly with silicon [129,130,131]. The characteristics of the Si-O-Si bond, large bond length (0.163 nm), bond angle (130°) and bond energy (445 kJ/mol) provide SR with superior performance properties including excellent chemical resistance, good electrical insulation capacity, high elasticity, excellent thermal, ultraviolet and ozone stability, high weathering resistance and very low glass transition temperature (T_g_ ≈ −120 °C), as well as biocompatibility [132,133,134,135]. SR foams combine the characteristics of silicone rubber and foam materials such as good resilience, high thermal stability, shape conformity, low density and light weight [136]. Silicone rubber foams exhibit enhanced temperature stability (–60 °C to 250 °C for long-term performance and up to 400 °C for short-term application), offering a wider range of operating temperature compared with any other organic rubber foams [137]. Luo et al. fabricated methyl vinyl silicone rubber foams with different spherical cell sizes via physical foaming (using spherical urea with different sizes as cell-forming agent) to study the relationship between the cell size and the mechanical properties [138]. They showed that silicone rubber foams with spherical cell diameter between 300 and 450 µm exhibited very good compression strength and compression stress-relaxation property. Liao et al. evaluated the effect of silica content, temperature and pressure on the viscoelastic properties of silicone rubber compounds prepared with scCO_2_ [139]. According to their results, the concentration of silica had an effect on both cell nucleation and cell growth because it acted as a heterogeneous nucleation agent and also increased the viscosity of the SR compounds. In addition, by reducing the saturation temperature (T_s_ = 40 °C), cell nucleation was prominent owing to the generation of a large volume of CO_2_ molecules. On the contrary, increasing T_s_ (60 °C and 80 °C) led to cell coalescence associated to rapid cell growth (controlling step). Altogether, they obtained SR foams with lower cell densities and larger cell sizes at high temperatures. Finally, high saturation pressure resulted in higher scCO_2_ plasticization effect and lower viscosity of the SR matrix leading to improved cell growth rates. Chen and coworkers developed 3D printed SR foams with trimodal porosity leading to outstanding properties, multifunctionality and multidimensional tunability (Figure 5) [140]. Printable viscoelastic inks were prepared by simply mixing sodium chloride (NaCl) with a polydimethylsiloxane (PDMS) precursor gel. The hierarchical porosity, produced by salt leaching and solvent removal, produced PDMS foams with unprecedented hyper-elasticity, extreme compressibility, cyclic endurance (near-zero irreversible shape deformation under extreme compression of 90% strain and 1000 cycles of large compression) and excellent stretching ability (maximum strain of 210%). The porous structure also worked as surface roughness to offer super-hydrophobicity. The modulus, elasticity, stretching ability and oil absorbance capacity were all tunable through ink formulation or computer aided drawing. Silicone rubber (VMQ) foams combined with chemically reduced graphene oxide (rGO) and 3-aminopropyltriethoxysilane functionalized graphene (FG) were prepared by Shi et al. to evaluate their rheological behavior, cell structure and mechanical properties [141]. The presence of rGO improved the strength of the matrix by two times, while FG increased it by six times. The main reason was the stronger interaction and excellent compatibility of FG with VMQ. The increased matrix strength also limited the shrinkage of the cell walls leading to VMQ/FG foams having larger cell size compared to VMQ/rGO foams. The mechanical results showed that FG can improve the tensile strength (130%) and elongation at break (140%) compared to VMQ/rGO foams. The lightweight and flexible SR/Ag plated hollow glass microspheres (HGM) were used for electromagnetic interference (EMI) shielding in composite foams with a gradient structure prepared by the combination of density induction and scCO_2_ foaming [142]. The composite foams have a conductivity of up to 279.3 S/m with only 0.51 vol.% Ag and a corresponding EMI shielding effectiveness (SE) reaching 30 dB when the thickness was only 0.7 mm. The multilayer structure design of the composite foam not only increased the EMI SE, but also did not increase the reflection. The three-layer composite foam presented an average EMI SE of 59 dB and a reflection power coefficient (R value) of 0.60 at a thickness of 2 mm. An excellent EMI SE stability of the composite foam was obtained due to the good flexibility of the SR matrix.

### 3.3. Type and Amount of Accelerators

In order to get useful properties, elastomers must be vulcanized. Despite the presence of various crosslinking process and systems, the rubber industries generally use sulfur or peroxide vulcanization systems for rubber curing [143,144,145,146]. Rubber vulcanization by sulfur may take several hours without any accelerator, but in their presence the reactions are completed in a few minutes. Furthermore, adding accelerators decreases the amount of sulfur needed leading to improved oxidation resistance [147,148]. For good control of the cellular structure and final properties, selection of a suitable accelerator type and content is crucial. In other words, during the curing process, the scorch time (the time required, at a fixed temperature, for the onset of crosslink formation) should be long enough to prevent complete rubber vulcanization before filling the mold cavity, but it should be short enough to increase the production rates [149,150]. That is why the vulcanization rate plays an important role because it affects both the cellular structure and crosslink density of the final foams.

The curing reaction of elastomeric foams based on sulfur and accelerators is very complex due to a large number of simultaneous reactions. So far, several accelerator systems have been used, but the most important can be divided into four groups based on their curing reaction scheme and chemical structure [151]. Firstly, aniline and thiocarbanilide (amine derivatives) were developed but these are not used anymore because of their toxicity. Secondly, dithiocarbamates and xanthates have high crosslinking rates, but almost no scorch time. Then, benzothiazoles and sulphenamides are the most extensively used today because of their excellent scorch properties. Finally, thiurams can be used as both ultra-accelerators and vulcanizing agents.

In general, the accelerator reacts with sulfur to produce monomeric polysulfides which are interacting with the rubber molecules to form polymeric polysulfides, as well as to reduce the time required for vulcanization [152]. The structure, properties and name of the most important accelerators are listed in Table 3.

The effect of sulphenamide accelerator (MBS, TBBS and CBS) on the curing kinetics and properties of natural rubber foams was investigated by Charoeythornkhajhornchai et al. [154]. The main information is listed in Table 4. Foams based on CBS show the fastest vulcanization rate with the lowest activation energy because CBS produces higher level of basicity of the amine species than any other accelerator, forming a complex structure with zinc ions as ligands in sulfur vulcanization. In addition, the fast curing rate of CBS leads to the formation of foams with the smallest bubble size, narrowest bubble size distribution and lowest cell density resulting in the lowest thermal conductivity and thermal expansion coefficient than other foams.

Despite the presence of an accelerator, zinc oxide and a fatty (stearic) acid are also added to activate the vulcanization reaction. Zinc oxide in combination with an acid forms a salt increasing the accelerator efficiency [155]. Ariff and coworkers examined the effect of different ratio of accelerator between TMTD and CBS on the properties of SMR-L/ENR-25 foams [156]. The results indicated that the induction time was increased with increasing CBS ratio because CBS is not an effective sulfur donor compared to TMTD. Higher CBS ratio also led to larger average cell size, thicker cell wall, lower expansion ratio (higher relative density) and higher compression set. The stress under compression increased with increasing CBS ratio since the resulting foams were highly affected by the matrix properties and higher relative density, i.e., a higher amount of stronger material in the cell walls.

### 3.4. Type and Amount of Fillers

Because neat elastomers have low strength and modulus, rubber foams can be reinforced by filler addition to significantly improve their stiffness and strength for practical use [157]. In fact, filler addition affects not only the mechanical properties, but also on the curing characteristics of the foams including the curing time, scorch time and aging properties. Different types of fillers have been used to reinforce rubber foams, but the most important ones are carbon black (CB), fibers, clay and silicates [40,158,159]. As expected, the level of property improvement depends on various parameters including particle size and geometry, degree of dispersion, aspect ratio and orientation in the rubber matrix, as well as the level of bonding (chemical, mechanical and physical interactions) with the rubber chains [160].

Vahidifar and coworkers reported that increasing the CB content (0–20 phr) in NR foams increased the cell density, compression modulus and hardness, but decreased the cell size [46]. Moreover, the sound absorption of NR foams was improved with higher CB content due to a stiffening effect of the matrix (cell walls). In another work, Vahidifar et al. used a hybrid reinforcing system containing organo-modified nanoclay (NC) and nanocarbon black (NCB) to determine the effect of NC content (0–10 phr) on the curing behavior, morphological and mechanical properties of NR foams including 10 phr of NCB [161]. The rheological results showed that increasing the NC content (0 to 10 phr) gradually changed the curing parameters such as 50% shorter scorch and curing time, higher curing rate, as well as higher initial and final torque. Furthermore, increasing the NC content resulted in higher tensile modulus and hardness, but decreased the foam resilience and elongation at break. Increasing the NC content from 0 to 5 phr led to the formation of foams with more uniform and smaller cells, while 7 phr NC produced a foam structure having two areas composed of different cell sizes (bimodal distribution). Nayak et al. found that increasing the CB content in ethylene-octene copolymer (EOC) foams resulted in lower curing time and foam expansion ratio [162]. On the other hand, their results showed that CB addition increased the cell density and decreased the average cell size. Higher number of cells was associated to a heterogeneous nucleation effect created by the CB surfaces, while smaller cell size was related to higher processing viscosity and faster curing rate inhibiting cell growth. Furthermore, Dalen et al. reported that kaolin addition (2–8%) can better improve the curing rate than calcium carbonate for polyurethane (PU) foams, but an excess of kaolin reduced the curing rate leading to processability problems [163]. Bashir and coworkers observed that the thermal stability of EPDM foams was improved with increasing CB content [164]. The viscoelastic and foaming behavior were decreased, while the curing and mechanical strength of the EPDM foams were improved. Phumnok et al. investigated the effect of filler types (CB, calcium carbonate and china clay) and their content (0 to 50 phr) on the physical properties of NR foams [159]. The results showed that for specific filler concentration, CB produced the highest tensile modulus and strength, as well as the highest torque difference and hardness, while china clay led to the highest elongation at break.

## 4. Chemistry of Foams

**Scheme 2:**
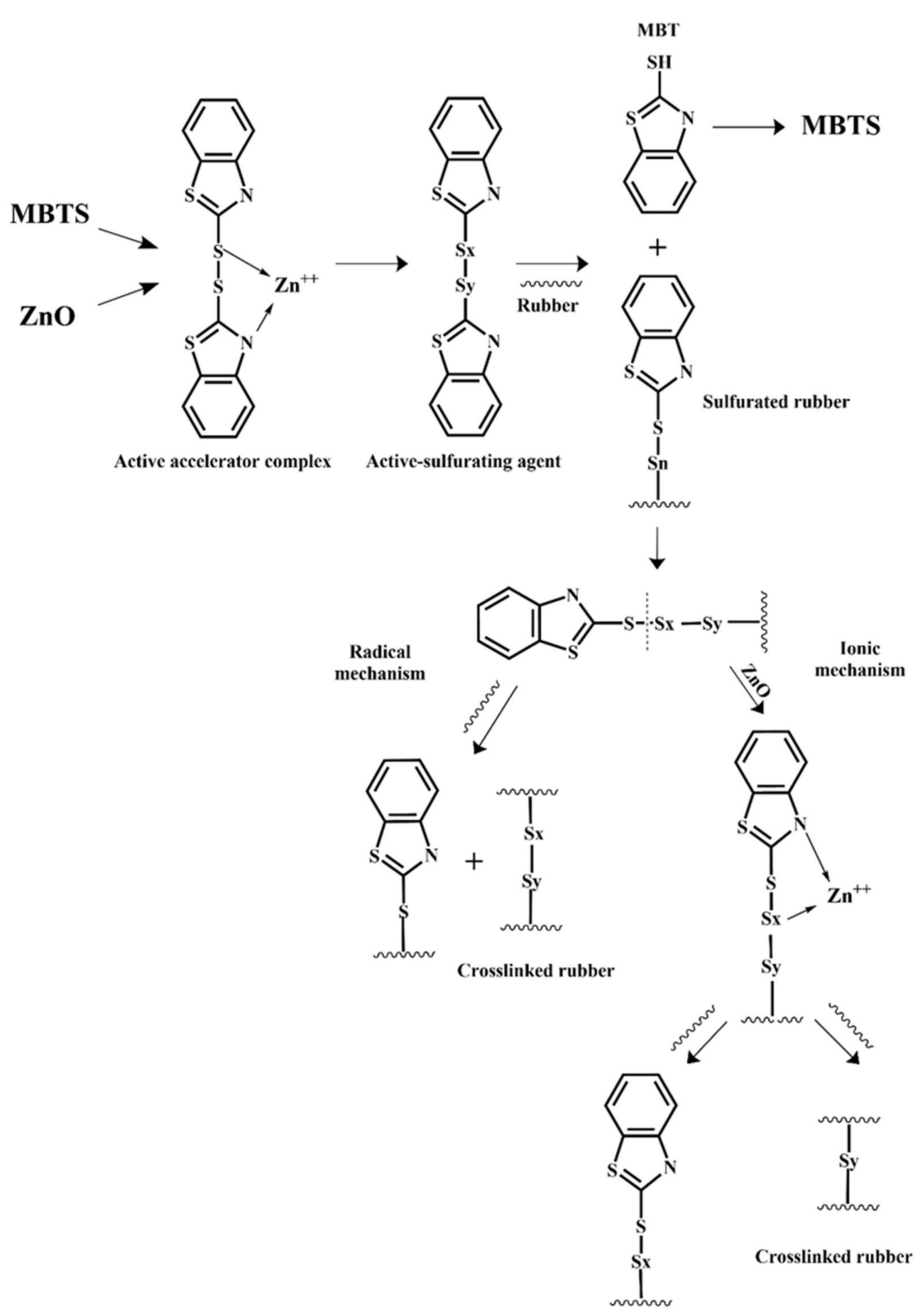
Sulfur curing process of NR in the presence of MBTS and ZnO as activator.

**Scheme 3:**
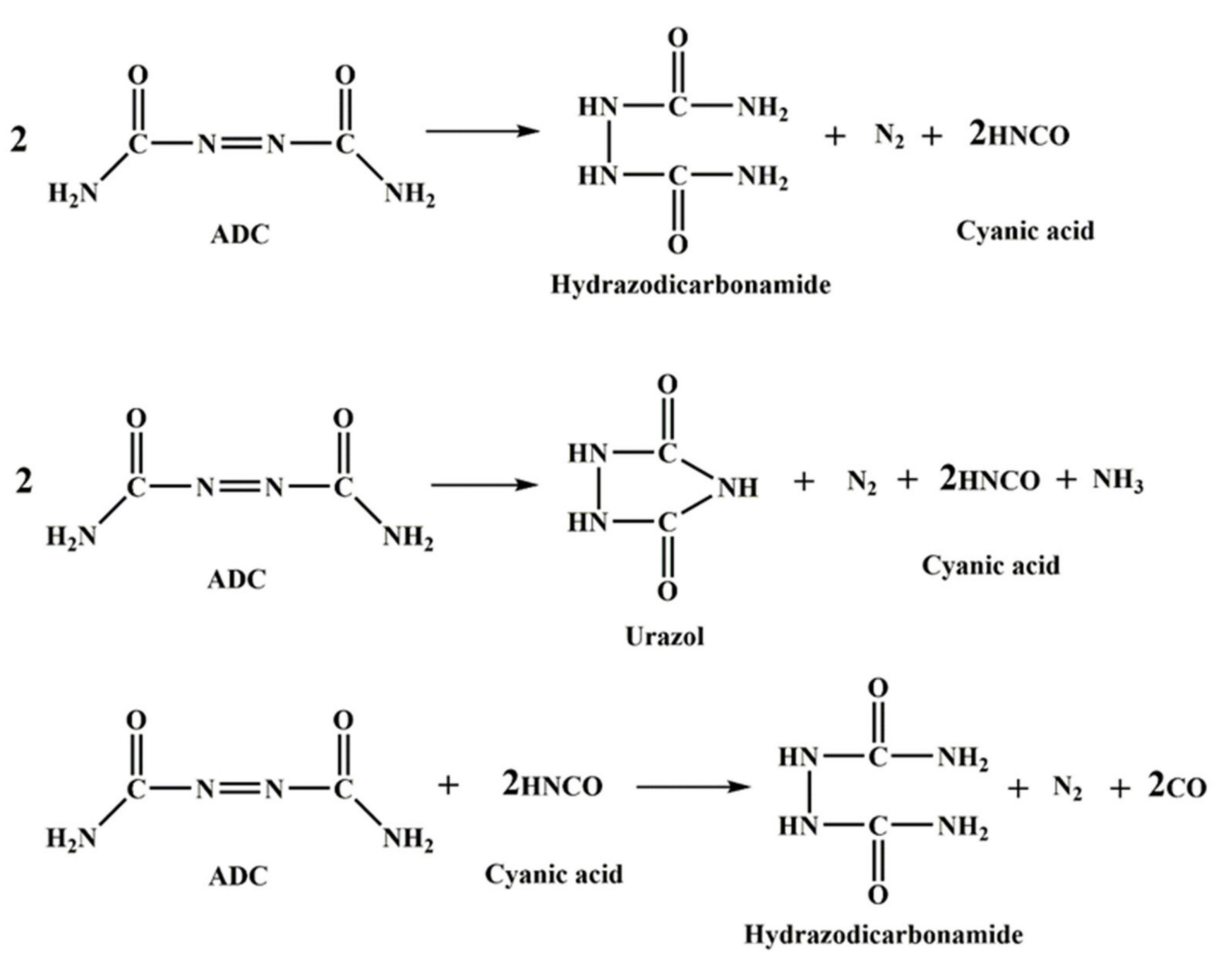
Decomposition mechanism of ADC [166].

In the production of rubber foams, two counteracting mechanisms are playing an important role to control the final foam properties by affecting the cell morphology: the foaming and crosslinking reaction. In fact, the curing reaction among the rubber molecules is necessary to stabilize the morphology and increase the viscosity of the foaming medium, but the effective amount of gas produced through the CFA thermal decomposition is the foaming driving force [165]. In general, curing reactions based on sulfur are performed in three steps [150]. The first step is the production of an active-sulfurating agent. In this step, the accelerator and the activator form the active accelerator complex. This complex reacts with the available sulfur and generates an active-sulfurating agent. In the second step, the active-sulfurating agent attacks the double bond of the weak allyl carbon inside the rubber chain forming the sulfurated rubber. Then, the sulfurated rubber attacks another double bond and the curing process continues. The third step is the postcuring step. The crosslink bonds produced in the previous step are mostly polysulfide (two to eight sulfur atoms in the crosslink bonds). Sulfur curing is also classified according to another method. In this classification, the chains can be cured with two ionic mechanisms and a radical mechanism. Studies have shown that in the presence of an accelerator, ZnO activator and stearic acid, both ionic and radical mechanisms occur. The mechanism of sulfur curing in the presence of MBTS and ZnO activators is shown in Scheme 2. On the other hand, the decomposition mechanism of ADC, as the most used CFA, is presented in Scheme 3 [166]. It can be seen that ADC decomposition is an auto-catalytic reaction [167]. Therefore, increasing the foaming agent content leads to faster gas generation rates.

These reactions can be controlled by appropriate selection of the processing conditions, such as temperature, formulation (concentrations) and components (molecules), involved in the foaming and curing systems [168]. As mentioned above, a balance between the kinetics of these two reactions is crucial to control the final foam morphology in terms of cell size and cell density. For example, if the rubber has slow curing rate and has not been given sufficient precure at the time of pressure release or once the foaming agent starts to decompose and releases the gas, the gas would either escape from the mass (low efficiency) or the generated cells will collapse due to a lack of material strength (cell wall rupture). On the other hand, if the curing is too far before the gases are released, these molecules will not be able to expand the “stiff” rubber (high viscosity and elasticity) resulting in a hard and high density foam containing a very low number of small cells [169].

**Figure 6:**
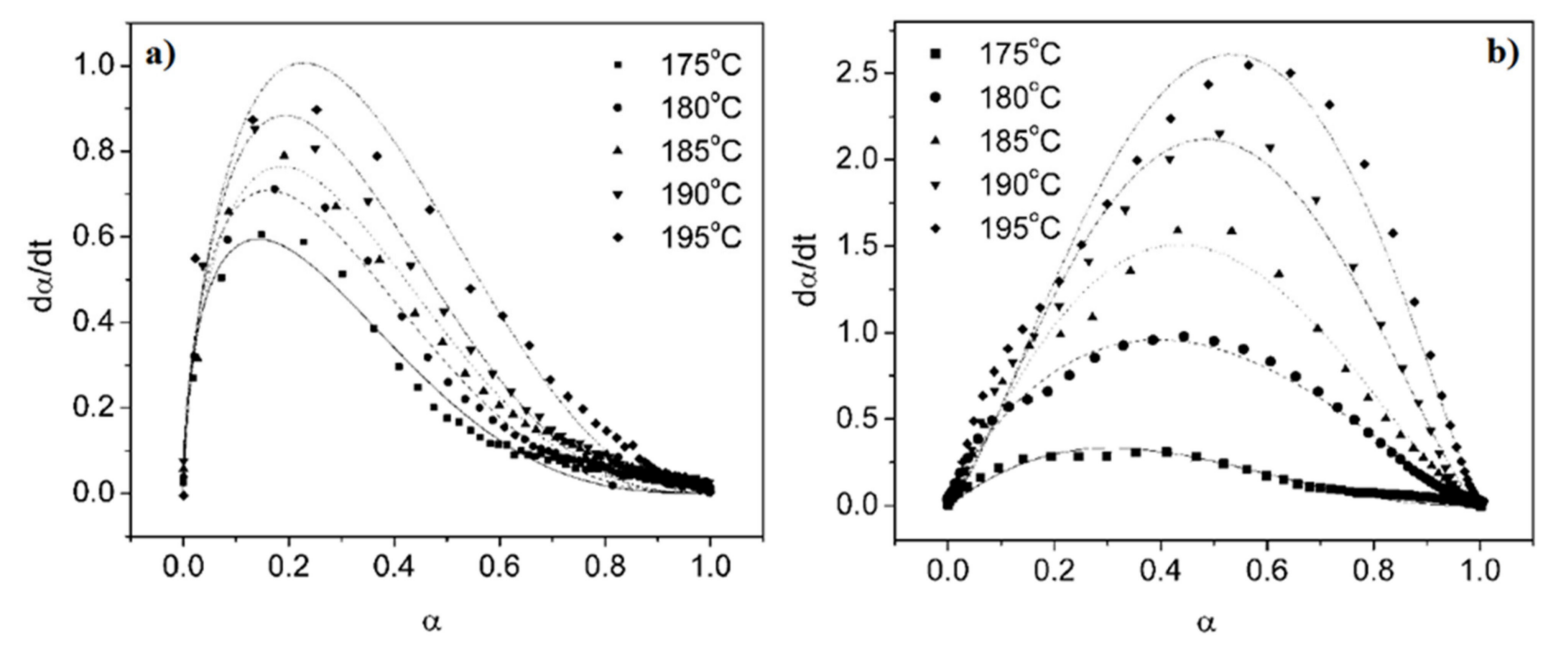
Reaction rate (*dα*/*dt*) as a function of conversion (*α*) for different temperatures: (**a**) vulcanization and (**b**) CFA decomposition [165].

In general, these two chemical reactions occur simultaneously making the system very complex [170]. For a better understanding of the chemical reactions between all the components, the degree of reaction can be indirectly measured. For example, measuring the rubber stiffness via a torque rheometer can be related to the crosslinking density inside rubber compounds. In addition, the pressure–time curve can be another way to get an accurate understanding of the CFA decomposition kinetics. The degree of cure (vulcanization) (*α_v_*) and the level of CFA decomposition (*α_D_*) can be calculated as [165]:

(5) $$a_{v}\left(t\right)=\frac{M_{t}-M_{i}}{M_{u}-M_{i}}$$

(6) $$a_{D}\left(t\right)=\frac{P_{t}-P_{i}}{P_{u}-P_{i}}$$

where *M_t_*, *M_i_* and *M_u_* are the torque at a given time t, initial torque (*t* = 0) and ultimate torque (equilibrium value) respectively, while *P_t_*, *P_i_* and *P_u_* are the pressure at a given time *t*, initially and equilibrium, respectively. By plotting the reaction rates (*dα*/*dt*) as a function of conversion (*α_v_* and *α_D_*) for various temperatures, Wang and coworkers proposed a mathematical model to relate the time and temperature for EPDM as (Figure 6) [165]:

(7) $$\frac{d_{\alpha }}{d_{t}}=K\left(T\right)f\left(\alpha \right)$$

where *K(T)* is a function of temperature and *f(α)* is a function associated with a phenomenological kinetic model and may have different forms based on the reaction mechanisms [171].

According to Figure 6, the shape of the reaction curves is temperature dependent as increasing the temperature results in higher reaction rates. Since the reaction rates are faster at higher temperature, the maximum conversion (*α*) for both rubber vulcanization and foaming agent decomposition are about 0.24 and 0.50 before the reaction rates decreased. The peak height for foaming agent decomposition was achieved at higher conversion than that of vulcanization for the same temperature. In a similar work, Vahidifar et al. found that the rate of conversion (*dα*/*dt*) for ADC decomposition is higher than that for IR vulcanization [93]. The rate of conversion was close to zero at both the beginning and the end of the conversions, while the maximum rate of conversion was larger than zero. These results are characteristics of auto-catalytic reactions and can be approximated by the Šesták-Berggren model as [172,173]:

(8) $$f\left(\alpha \right)=\alpha^{m}{\left(1-\alpha \right)}^{n}$$

Combining Equations (7) and (8) gives:

(9) $$\frac{d_{\alpha }}{d_{t}}=K\left(T\right)\alpha^{m}{\left(1-\alpha \right)}^{n}$$

Moreover, the *K*(*T*) function depends on the activation energy (*E_a_*) which can be written in the form of an Arrhenius equation as:

(10) $$K\left(T\right)=A\;exp^{-E_{a}/RT}$$

Linearization of the Arrhenius equation yields:

(11) $$\ln K\left(T\right)=lnA-E_{a}/RT$$

where *A* is the Arrhenius constant, *R* is the universal gas constant and *T* is the absolute temperature. According to Equation (11), *E_a_* and *A* can be calculated from the slope and intercept of the linear form of the Arrhenius plot: ln *K*(*T*) vs. 1/*T*. The results show that *E_a_* for ADC decomposition (*E_a_* = 182 kJ/mol) is higher than that of rubber curing (*E_a_* = 79 kJ/mol), which confirmed that by increasing the temperature of foaming agent decomposition accelerates more than the rubber vulcanizing reaction [93,165].

## 5. Foaming Technology

There are three main techniques for rubber foam processing: batch foaming, extrusion and injection molding [16,174,175]. Batch foaming is mostly used for bulk products, fundamental studies, laboratory investigations and small production runs, while the other methods are often used for large-scale production at an industrial scale.

### 5.1. Batch Foaming

**Figure 7:**
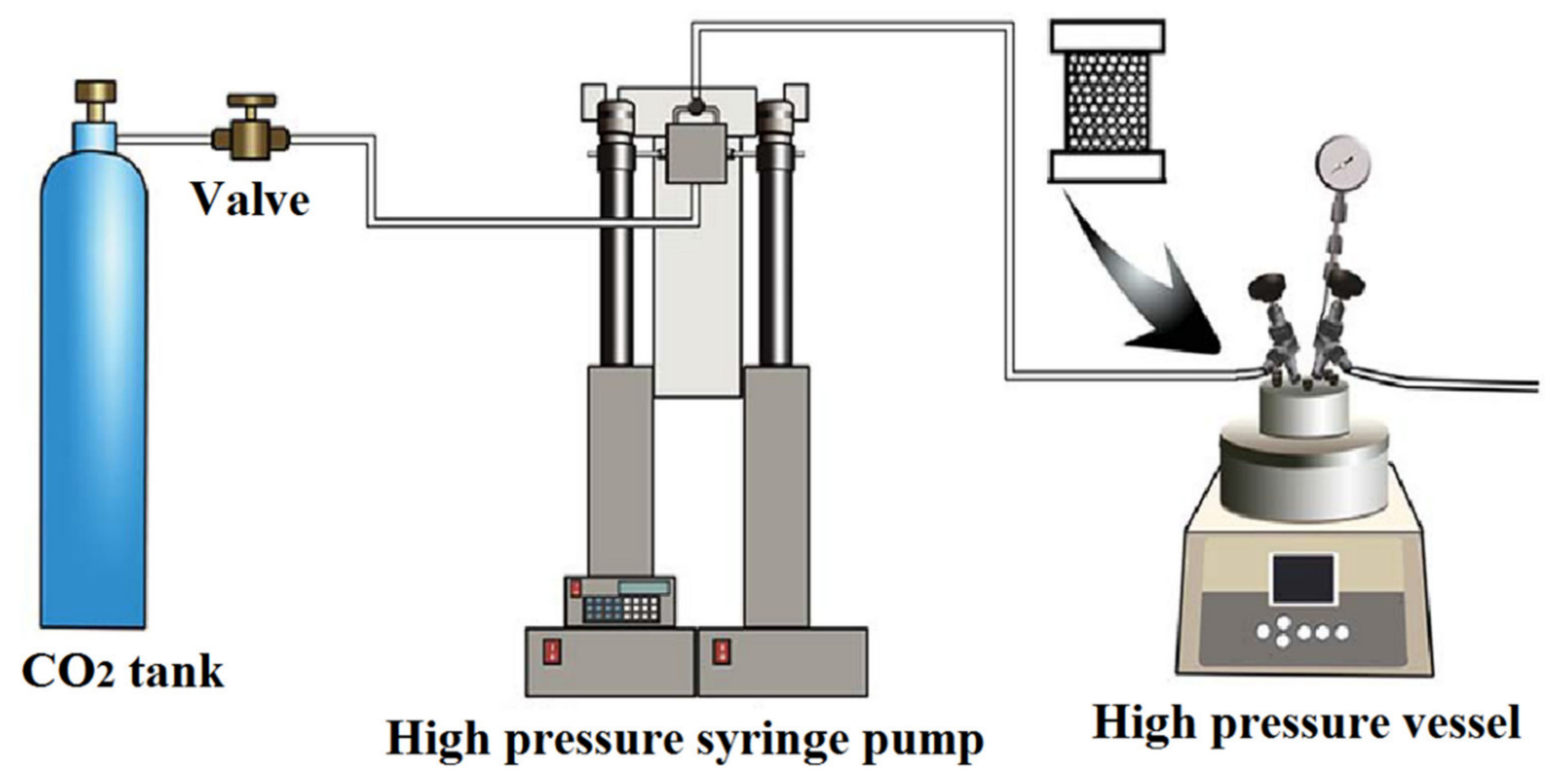
Schematic illustration of the batch foaming process using CO_2_ as a PFA [176].

Batch foaming, also known as “solid state foaming”, as the name implies is a batch-wise process in a closed system. Firstly, the neat rubber has to be masticated to decrease the molecular weight and viscosity allowing a simple and homogeneous mixing of additives. Then, all the ingredients, except for the curing agent, are added and mixing is continued for several minutes. Finally, the curing agent is fed and mixed. The prepared compound is stored overnight at room temperature to release residual stress of the rubber molecules during mixing. Then, the compound is placed in a mold and hot pressed at a defined pressure, temperature and time depending on the curing and foaming kinetics as described above. Figure 7 presents a schematic representation of the batch foaming process using a physical foaming agent [176]. The polymer pellets are placed in a stainless-steel mold with micro-nozzles. Then, the mold is put inside a high-pressure vessel (autoclave). After being saturated (specific conditions), the foamed parts are obtained by a rapid depressurization (thermodynamic instability due to the creation of a supersaturated state leading to cell nucleation). The samples produced are usually in the form of circular discs or rectangular/square plates. The sample thickness is usually between 5 mm and 50 mm which is a very important dimensional factor related to gas diffusivity.

### 5.2. Foam Extrusion

**Figure 8:**
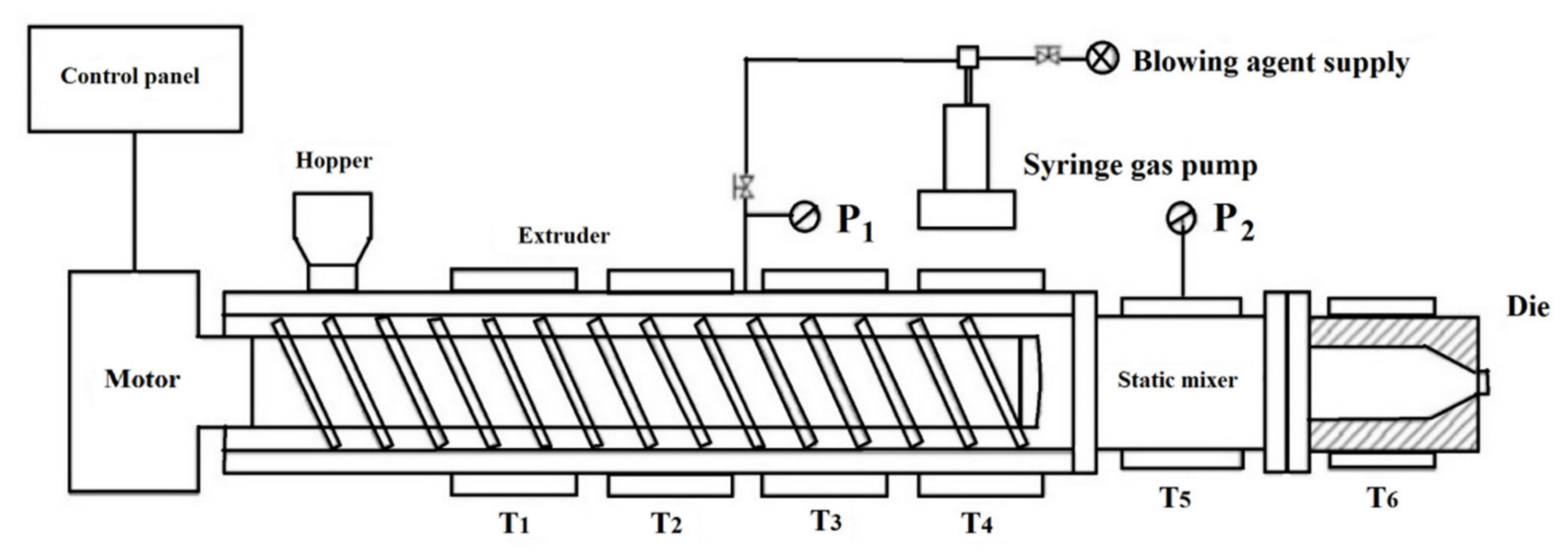
Schematic representation of an extrusion foaming system based on PFA [189].

The term extrusion is derived from Greek meaning to ‘‘push out” [177]. Extrusion is a continuous process composed of an extruding unit and a die-shaping unit (Figure 8) [178]. The rubber compounds in strip or pellet forms are fed into the extruder through the feed hopper and drawn into the rotating screw. The screw speed and the barrel temperature profile are controlling the flow rate and temperature [179]. As mentioned before, the crosslinking and foaming reactions are strongly dependent on temperature. Hence, the barrel temperature and the screw speed are two important parameters also affecting the material self-heating via viscous dissipation [180]. On the other hand, the surface quality is an important feature of rubber foam profiles. During extrusion, the high shear stresses in the extrusion die can exceed the tear strength of the rubber compound, resulting in surface defects (cracks) of the extrudate. In addition, the foaming reaction during vulcanization can produce small bubbles on the surface layer resulting in undesired roughness. Thus, the temperature profile and screw speed control the vulcanization and extrusion process, especially for extrusion foaming [181]. In most cases, the final part of this process is calibration and cutting of the extruded profiles. The products of extrusion foaming include insulation foam boards, pipes and other products which can be made by profile extrusion [182,183]. Hopmann and coworkers used extrusion to produce EPDM and NBR foams using water as a physical foaming agent [179]. They found that due to dissipative heating and heat conduction from the extruder into the rubber, the tendency to generate surface defects (cracks and bubbles) increased. Park et al. stated that the cell density of foams produced via extrusion depends on the of rate pressure drop [184,185]. When the pressure drop occurs, the gas molecules in solution either lead to the growth of pre-formed cells or generate new nuclei to lower the system’s free energy. However, increasing the pressure drop leads to the nucleation process being favoredinstead of existing cell growth. Therefore, higher cell density and lower cell size is obtained [186]. The effect of pressure and shear stress on cell nucleation in foam extrusion was studied by Lee, who found that shear rate has a direct effect on cell nucleation [187]. Chen and coworkers stated that the relation between pressure drop rate and shear stress is very critical when the saturation pressure is low [188]. This is due to the lack of sufficient driving force for cell nucleation. In general, shear stresses have a higher effect on cell nucleation compared to the pressure drop rate. The main reason is that the volume of gas molecules needed for cell nucleation decreases by imposing shear stresses.

### 5.3. Foam Injection Molding

Conventional injection molding (CIM) and foam injection molding (FIM) are methods used for unfoamed and foamed products, respectively. In general, injection molding is composed of two parts: injection unit and clamping unit [190,191]. After adding the rubber compounds into the screw in the shape of pellets through the hopper, the injection unit melts the rubber compounds and maintains the injection pressure during mold filling. The function of the clamping unit is for precise mold opening and closing with an appropriate clamping force, cooling, and sample demolding.

The reciprocating screw motion in the injection molding is the main difference with extrusion. In foam extrusion, the screw rotation continuously pushes the melt forward and out of the extruder through the die. However, in injection molding, the screw rotates and moves backwards due to the accumulation of a pool of gas-loaded melt at the tip of the screw. Thereafter, the gas-filled melt is injected through the nozzle (channel) into the mold cavity at high temperature and pressure. In order to compensate for shrinkage, a holding step is applied to further fill-in the mold after the melt injection process is completed. The mold is then opened, and a rapid pressure drop occurs (atmospheric pressure). Cell growth occurs after cell nucleation, followed by expansion and stabilization of the cellular foam with an unfoamed layer (skin) which is dependent on the degree of rapid cooling of the mold [192]. Altogether, this method decreases the cycle time and improves the quality of foaming as much lower pressure and clamping force is needed leading to lower production cost [41]. Injection molding is the most promising process because it can produce products of variable geometry and size ranging from microprocessor sockets to automobile door modules [183]. Moreover, foamed rubber molded parts are used in several applications due to their low material use, great dimensional stability, low back pressure, wide range of mechanical properties and high stiffness-to-weight ratio [56,74,193,194,195,196]. Today, the process is highly controlled and understood via several tools like numerical analysis and process parameter optimization [197]. Table 5 presents a comparison between batch foaming, extrusion foaming and injection molding foaming to manufacture rubber foams.

## 6. Foam Morphology

As mentioned before, two categories of parameters are affecting the physical and mechanical properties, as well as the microstructure of rubber foams. The foaming process consists of two fundamental steps: nucleation and growth. After decomposition of the chemical foaming agent or injection of a physical foaming agent, a new phase (bubble phase) is generated that is called the nucleation step. In the growth phase, the bubble nuclei grow into final bubbles or cells. Both steps are affected by several physical properties such as viscosity, solubility, diffusivity, surface tension and glass transition temperature. Since the foaming and curing processes are performed simultaneously, controlling the nucleation and growth steps is very complex. A proper selection of the formulation (foaming and curing systems) and processing parameters (pressure, temperature, time, etc.) is crucial to obtain a specific morphology and structure of rubber foams [198,199]. In the next section, the effect of these processing parameters (foaming and curing) is discussed in detail. 

### 6.1. Thermodynamics of Nucleation

Foaming is an intricate process involving some thermodynamics phenomena, kinetics and transport phenomena [200]. The complete control of all the aspects related to cell morphology is a challenging issue in the rubber foam industry. Since cell nucleation and growth are the main steps governing the final foam structures and quality, a better understanding of the mechanisms involved in these processes is important for process optimization and to control the final foam structure.

During foaming with a chemical foaming agent, the decomposition of the solid CFA (powder) produces some residues remaining in the rubber matrix. This can be advantageous (heterogeneous nucleation effect) or not (contamination/properties loss). Since a small amount of gas molecules is used for the nucleation step, achieving a product with high cell density and uniform cell morphology is difficult. Therefore, the main part of gases produced is used for cell growth. On the other hand, the viscoelastic behavior of the matrix plays an important role in rubber foaming: low viscosity leads to cell collapse and cell coalescence, while high elasticity restricts the bubble nucleation and growth steps. Therefore, a balance must be achieved as a minimum level of pre-crosslinking is helpful to optimize the foaming behavior, but not too much to impede it [201].

**Figure 9:**
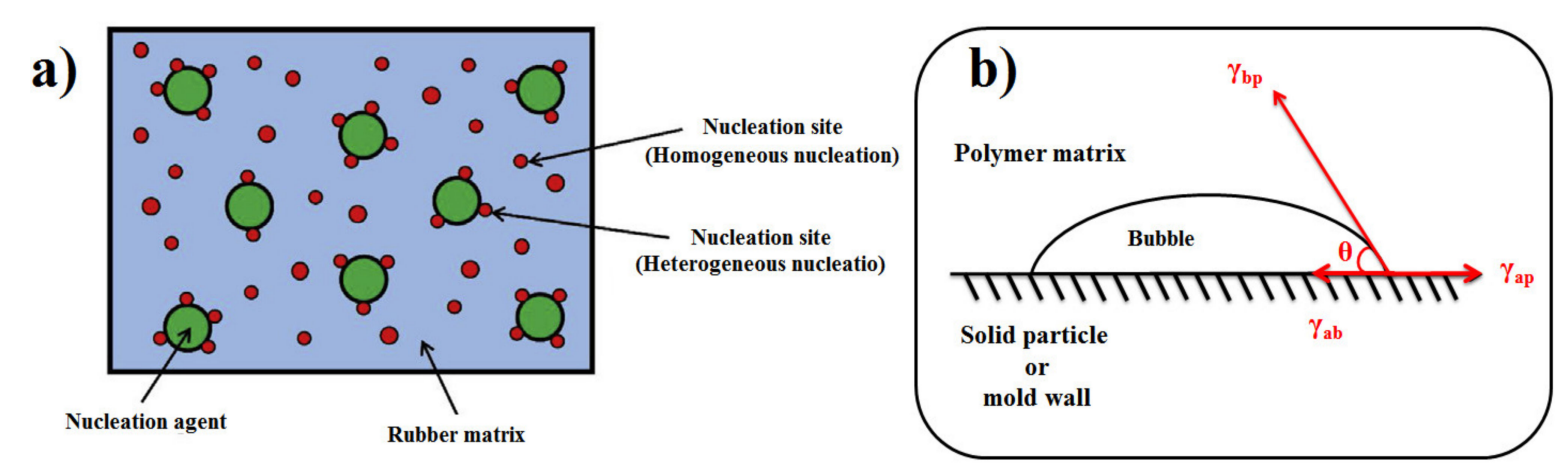
(**a**) Schematic diagram of homogeneous and heterogeneous nucleation during foaming in the presence of a nucleation agent [73] and (**b**) interfacial tensions of the optimum bubble shape for heterogeneous nucleation.

The classical nucleation theory is mostly used to model the bubble nucleation inside the rubber matrix [202,203]. This theory was originally developed for liquid droplet formation from a vapor, but it can also be applied to the inverse case of interest in polymer foaming applications. However, several approximations are needed for using this method. Firstly, only homogeneous nucleation takes place and the interfacial tension between the gas nuclei and the matrix is considered equal to the surface tension [204,205]. The CFA decomposition produces the gas nuclei inside the rubber matrix which can be attributed to both homogeneous and heterogeneous nucleation. The gathering of gas molecules inside the rubber matrix leads to homogeneous nucleation. On the contrary, heterogeneous nucleation occurs when the nuclei are formed on the boundaries between two phases like on the surface of solid particles (fillers or impurities), on preexisting gas cavities, or between areas of different density due to the dispersed crystallites or due to insufficient thermal processing during rubber molding. However, the activation energy of homogeneous nucleation is much higher than that of heterogeneous nucleation, so the latter is more favorable. Figure 9a presents a schematic diagram of the homogeneous and heterogeneous nucleation during foaming in the presence of nucleation agent. Although this theory has been able to describe the effect of pressure and temperature on nucleation (qualitative analysis), it has been shown to be inappropriate to describe with precision the bubble nucleation step inside polymer matrices [206]. The generation of bubbles via homogenous nucleation occurs randomly and spontaneously without any foreign bodies or additives (like for heterogeneous nucleating agents). In fact, a local fluctuation in temperature and/or pressure leads to homogenous nucleation. On the contrary, heterogeneous nucleation does not occur spontaneously. It is triggered by the presence of a second phase (additives) acting as nucleation points decreasing the nucleation energy and increasing the nucleation rate [207]. The presence of additives lowers the Gibb’s free energy, but this depends on the particle surface topography (shape factors).

The homogeneous nucleation of gas nuclei inside a metastable polymer matrix with dissolved additives can be defined by the classical nucleation theory with modifications accounting for changes in free volume and interfacial energy [208]. The production of a gas bubble in a polymer through a reversible thermodynamic process has an excess energy associated to:

(12) $$\Delta G_{\mathrm{hom}}=-V_{b}\Delta P+A_{bp}\gamma_{bp}$$

where *V_b_* is the volume of the bubble nuclei, Δ*P* is the pressure difference between the inside and outside of the gas bubble, *A_bp_* is the surface area of the bubble and *γ_bp_* is the interfacial tension associated to the matrix-bubble interface. This excess energy can be minimized by a suitable choice of the bubble shape. If *γ_bp_* is isotropic, this shape will be spherical with a radius *r* and Equation (12) becomes [209,210]:

(13) $$\Delta G_{\mathrm{hom}}=-\frac{4\pi r^{3}}{3}\Delta P+4\pi r^{2}\gamma_{bp}$$

When Δ*G* is plotted against *r*, the curve shows a maximum at a critical radius *r_c_* given by:

(14) $$\frac{d\Delta G}{dr}=0\;\Rightarrow \;r_{c}=\frac{2\gamma_{bp}}{\Delta P}$$

The maximum value of Δ*G* for homogeneous nucleation is obtained by substituting Equation (14) into Equation (13) to give:

(15) $$\Delta G^{*}{}_{\mathrm{hom}}=-\frac{16\pi \gamma^{3}}{3\Delta P^{2}}$$

As mentioned before, heterogeneous nucleation occurs on the mold walls or/and by addition of an insoluble phase (solid particles) to the solution as illustrated in Figure 9b. The nucleus has the shape of a spherical cap to minimize the energy and the ‘wetting’ angle *θ* is given by a force balance at the interface. The relation for stress equilibrium is [211]:

(16) $$\cos\theta =\frac{\gamma_{ap}-\gamma_{ab}}{\gamma_{bp}}$$

where *γ_ap_*, *γ_bp_* and *γ_ab_* are the interfacial tension between the solid particle-polymer matrix, bubble-polymer matrix and solid particle-bubble, respectively. The heterogeneous critical nucleation energy at equilibrium state is given by:

(17) $$\Delta G^{*}{}_{\mathrm{het}}=-V_{b}\Delta P+A_{bp}\gamma_{bp}+A_{ab}\gamma_{ab}-A_{ap}\gamma_{ap}$$

(18) $$\Delta G^{*}{}_{het}=\left\{-\left(\frac{4}{3}\right)\pi r^{3}\Delta P+4\pi r^{2}\gamma_{bp}\right\}S\left(\theta \right)$$

(19) $$S\left(\theta \right)=\left(1/4\right)\left(2+cos\theta \right){\left(1-\cos\theta \right)}^{2}$$

where *V_b_* is the volume of the bubble, while *A_bp_*, *A_ab_* and *A_ap_* are the bubble-polymer matrix, solid particle-bubble and solid particle-polymer matrix interfaces, respectively. The differentiation of Equation (18) provides an expression for the critical nucleus radius r* as:

(20) $$r^{*}=2\gamma_{bp}/\Delta P$$

so,

(21) $$\Delta G^{*}{}_{het}=\frac{16\pi \gamma_{bp}^{3}{}_{hom}}{3\Delta P^{2}}S\left(\theta \right)$$

(22) $$\Delta G^{*}{}_{het}=\Delta G^{*}{}_{hom}S\left(\theta \right)$$

Because *S(θ)* has a value always smaller than 1, the heterogeneous critical nucleation is smaller than for the homogenous one according to Equation (22) [207].

### 6.2. Foaming Process

The rubber foaming process can be decomposed into three steps: creating small discontinuities or cells in a fluid or polymer phase (nucleation), allowing these cells to grow to a desired volume (growth) and stabilizing this cellular structure by physical or chemical means (stabilization). Temperature, pressure, and number of expansion step (one-stage or two-stage) are important foam processing factors affecting these three steps controlling the microstructure, density, mechanical and physical properties of the final foams. The next section reports on the effect of these three factors.

#### 6.2.1. Temperature

**Figure 10:**
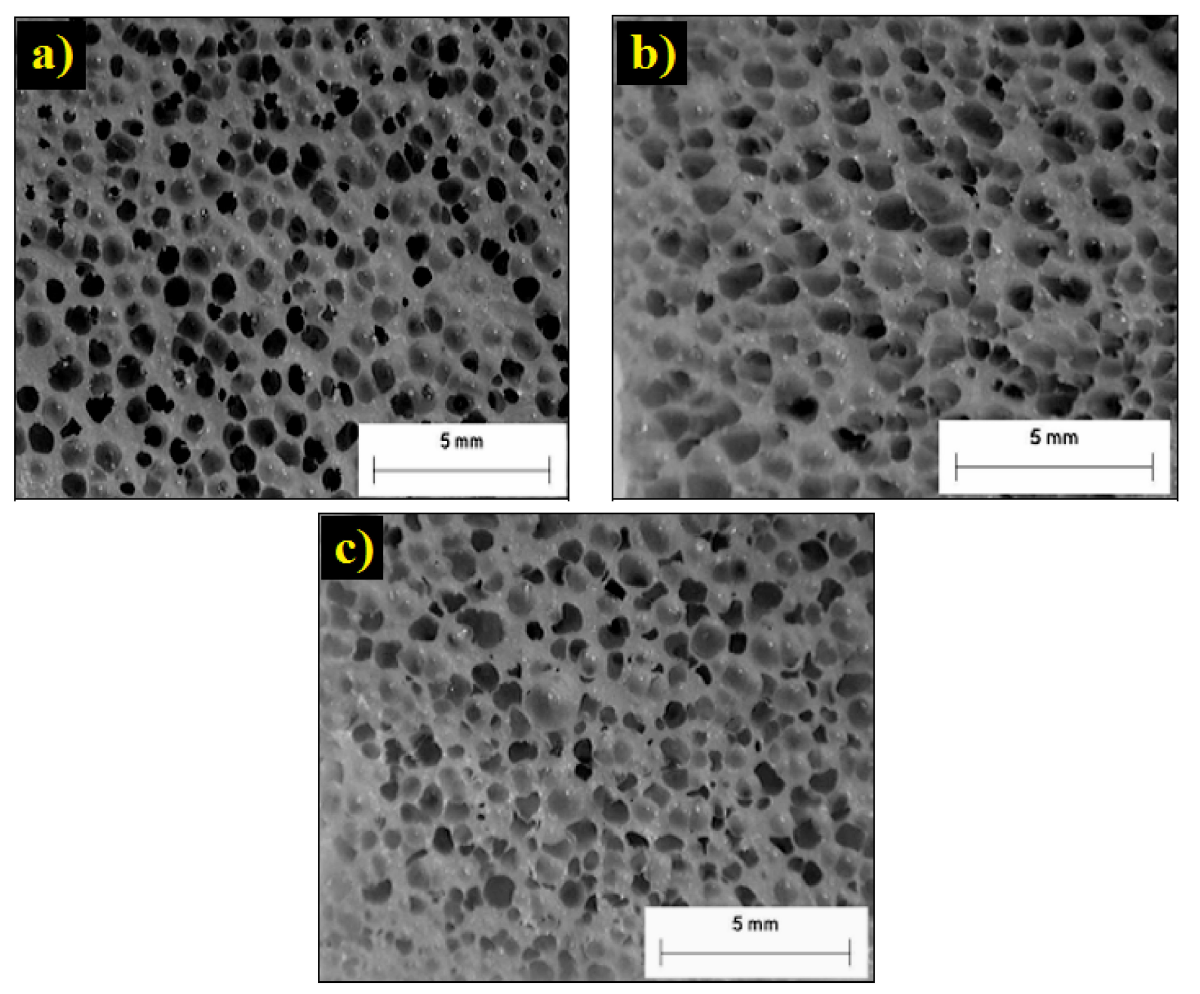
Morphology of EPDM foams produced under different temperatures: (**a**) 140 °C, (**b**) 150 °C and (**c**) 160 °C [199].

Temperature mainly controls the melt viscosity which has a direct effect on cell size and cell density (foam density) [212]. For a successful foaming process, the temperature must be low enough to increase the viscosity and have good nucleation, while cell expansion requires low viscosity (high temperature) for easier expansion, but not too much to prevent cell collapse/coalescence. Vahidifar et al. reported that the cell structure evolution is related to a competition between two phenomena. The first one is the volume and the rate of gas released from the foaming agent decomposition as a foaming driving force resulting in high cell nucleation and growth, while the second one is an increased resistance of the matrix (viscosity) due to the crosslinking reaction limiting further expansion leading to stabilization [169]. Hence, the optimum foaming temperature can be determined due to a competition between foaming and curing processes. Zakaria and coworkers investigated the effect of foaming temperature (140, 150 and 160 °C) on the cell morphology of EPDM foams [199]. They used sodium bicarbonate as CFA which has a decomposition temperature in the range of 120–200 °C. Increasing the foaming temperature resulted in foams with larger cells as indicated in Figure 10. The foam prepared at 140 °C had a more uniform cell structure with discrete cells compared to the cell morphology produced at 150 °C and 160 °C. In addition, the foam prepared at lower temperature had thicker cell walls than the foams made at high temperatures due to the smaller cell produced at lower temperature. However, the cell size at 160 °C is slightly smaller than at 150 °C. The reason is that at higher foaming temperature the crosslink rate is significantly increased preventing complete expansion. At higher foaming temperature, higher volume of gas is generated by the CFA promoting the cell expansion and subsequently the cells will come into contact and combine with each other to produce larger cells (coalescence). The physical and mechanical properties of NR foams were investigated as a function of the foaming temperature (145–155 °C) [213]. The density of the foamed NR decreased with increasing foaming temperature. The NR foamed at lower temperature (145 and 150 °C) had lower densities than those foamed at higher temperature (155 °C) because the cell density inside the rubber matrix increased with increasing temperature. They also found that the optimal temperature for vulcanization and foaming was 155 °C for these NR foams. The tensile strength was found to decrease with increasing temperature because of the lower density, indicating better foaming efficiency. Moreover, the tear strength and tensile modulus continuously decreased with increasing temperature.

**Figure 11:**
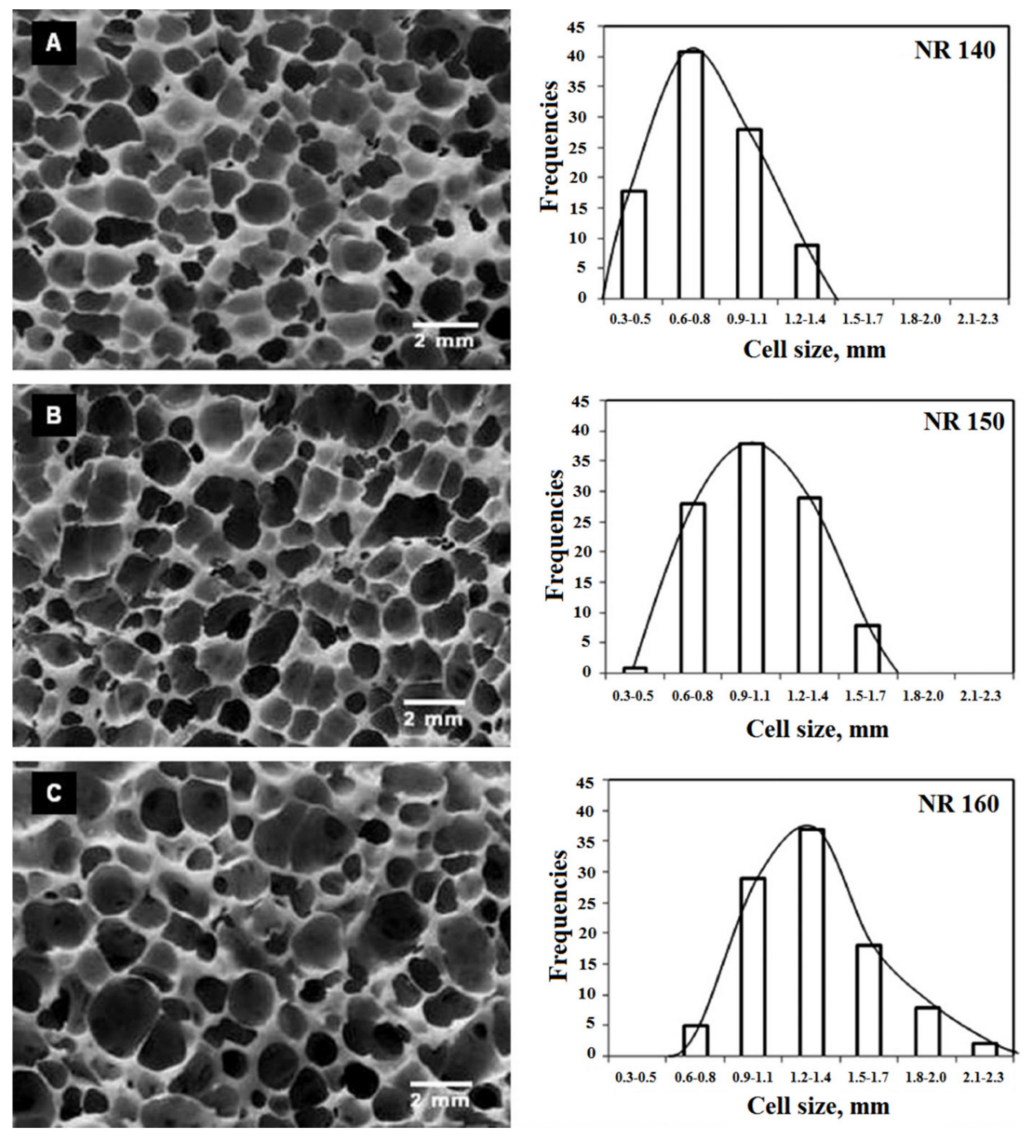
Morphology and cell size distribution of NR foamed under different temperature: (**A**) 140 °C, (**B**) 150 °C and (**C**) 160 °C [77].

Najib and coworkers reported that a higher foaming temperature generates a higher gas pressure stretching more the cell walls [77]. This expansion of the cell walls led to a relatively larger cell structure resulting in lower foam density. Hence, the amount of solid phase is reduced (per unit volume), and less crosslinking occurs. Figure 11 shows the variation of the foam morphology and cell size distribution as a function of foaming temperature. Increasing the foaming temperature not only improved the cell size distribution, but also extended the cell size distribution range. NR foams produced at 140 °C had a more uniform cell size distribution compared to the foams manufactured at 150 °C and 160 °C.

#### 6.2.2. Pressure

Another important factor affecting the morphology of rubber foams is the pressure used to form/mold a sample. The effect of foaming pressure on the morphology and mechanical properties of NR foam using DPT was studied by Kim and coworkers [214]. Increasing the foaming pressure resulted in higher foam density (lower expansion ratio) and lower foaming efficiency. The mechanical properties of NR foams, such as tear strength and hardness, gradually increased with increasing foaming pressure, while the elongation at break decreased. 

Since the most widely used physical foaming agent is scCO_2_, as an environmentally friendly alternative, the saturation pressure is strongly affecting the cellular microstructure [146,215]. Tessanan and colleagues examined the effect of pressure (0, 8.5, 10.5 and 12.5 MPa) on the structure of NR foams using scCO_2_ [73]. Their results showed that the average cell size decreased (less than 10 µm) and the cell size distribution was relatively narrower by increasing the saturation pressure. The main reason is that the activation energy for nucleation is lower with increasing pressure (Equation (15)) and a higher amount of gas molecules (saturation/solubility) is available to generate the nuclei. This large amount of cell nuclei density simultaneously growing in a restricted volume leads to a higher number (cell density) of smaller cells (average diameter) and a more uniform cell structure (narrower distribution) [176]. In another work, a series of microcellular high temperature vulcanizates (HTV) silicone rubber foams were produced using CO_2_ [216]. It was shown that CO_2_ diffusivity in the HTV silicone rubber also increased with saturation pressure. Moreover, the cell diameter decreased, and the cell density increased with increasing saturation pressure because higher pressure led to more CO_2_ absorbed by the matrix (higher gas concentration). Therefore, this high CO_2_ concentration increased the nucleation ability leading to a cell diameter reduction, i.e., since the amount of gas is fixed, a higher number of smaller cells is produced.

#### 6.2.3. Number of Stages

The expansion of rubber foams can be performed via a single stage or multiple (usually two) stages [217,218]. Basically, the first stage of a two-stage expansion is the same as for the single-stage process, but after pressure release (mold opening or outside an extrusion die), the expandable compound is immediately transferred to a circulating hot air oven at a higher temperature [83]. The one-stage process is known as a pressure-induced method, while the two-step process is usually referred to as a temperature-induced method [41].

In the one-stage foaming process with a physical foaming agent, the rubber matrix is firstly saturated with a PFA in an autoclave to reach equilibrium after a saturation time which is a function of pressure, temperature and PFA. Then, cell nucleation and growth take place by rapid depressurization of the system to atmospheric pressure. To stabilize the final morphology, postfoaming occurs during the cooling process. For a CFA, the compound (matrix mixed with the foaming agent) is placed in a mold and compressed (electrically heated compression molding press) at a fixed temperature and pressure for a specific time. Mold opening releases the pressure and cooled down to room temperature to fix the cell structure [219].

In the two-stage process with a PFA, the sample is taken out from the autoclave after reaching the equilibrium saturation. It should be noted that the saturation process of temperature-induced method usually takes place at lower temperatures compared to the pressure-induced method. In the second stage, the saturated sample is placed in a hot bath (oil) set at a defined temperature for a specific time to induce cell nucleation and growth. The sample is then removed from the bath and immersed in a cooling bath (water or other solvent). If the expansion is based on a CFA, after preheating and producing the compounds in a hot press, the sample is placed in a hot air oven for simultaneously curing and foaming. It is finally cooled down to room temperature to stabilize the cell structure. 

In general, the two-stage method is not a good method to produce foams with very precise and specific density. The method is also not very effective for producing foams with complex and precise shapes or dimensions. It is clear that foams expanded via the one-stage method have shorter processing times, which will increase the production rate [46]. For this reason, most products are made by the one-stage method and have lower production costs. Moreover, this method can produce a constant expansion ratio (foam density) along with easier control of the cell density/size leading to more uniform mechanical properties [52].

### 6.3. Curing Process

Crosslinking or curing, which is the formation of covalent, hydrogen or other bonds between rubber chains, is a very widely used technique to modify the rubber properties [220]. Initially, Charles Goodyear reported in 1839 a commercial method of crosslinking [221]. His process, vulcanization, was firstly successfully used in 1841 [222]. He found that heating a rubber with sulfur produced a reaction modifying the chemical structure of rubbers to improve their physical and mechanical properties. In general, curing with sulfur transformed a “viscous” rubber into a highly “elastic” elastomer [223].

**Figure 12:**
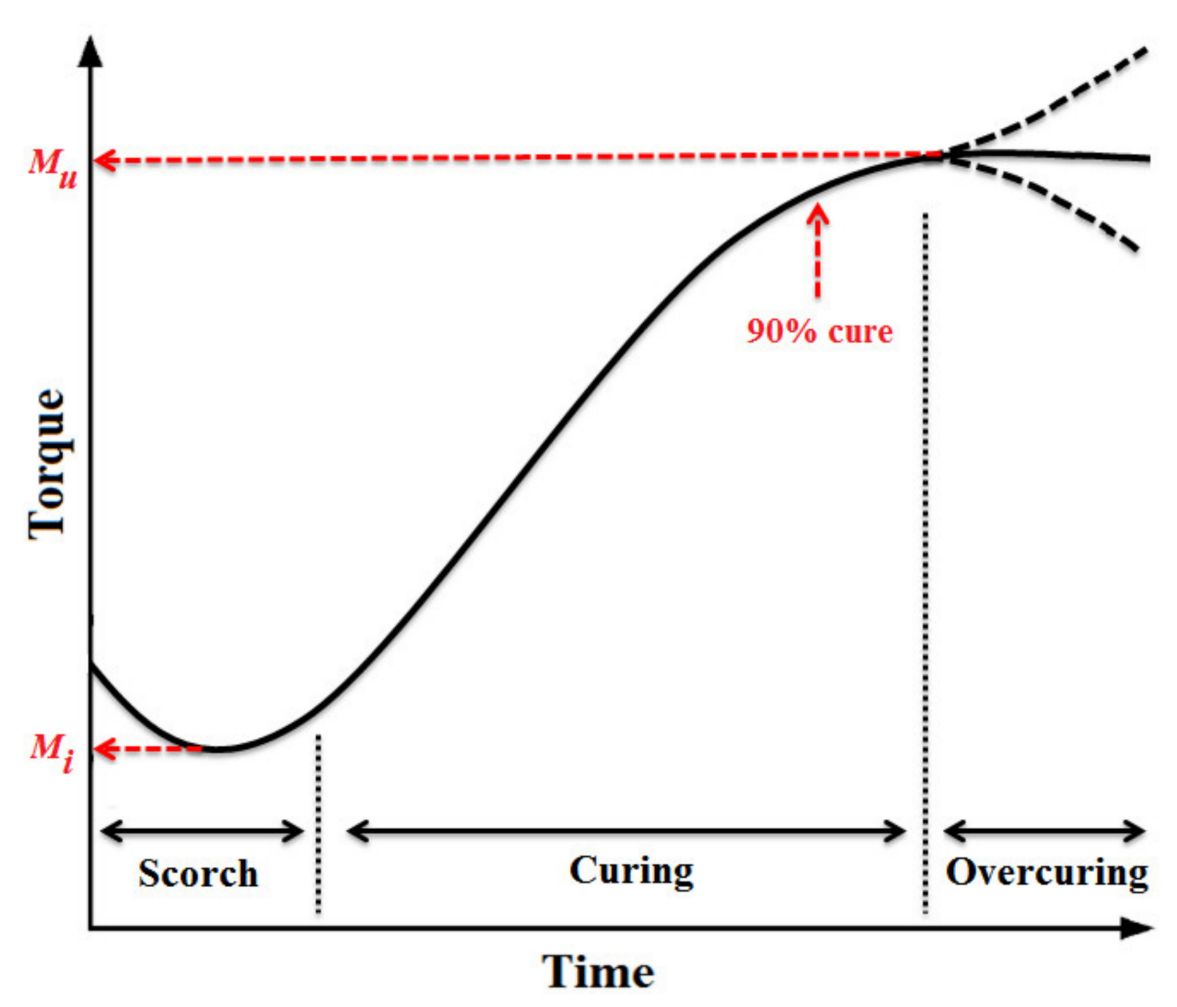
A typical rubber vulcanization curve (accelerated sulfur).

The curing of rubber foams is mainly studied by oscillating disk rheometry (vulcanizing curve) and differential scanning calorimetry (DSC) [173]. Rheometry follows the change in torque as a function of time, while DSC assumes that the heat of reaction is only due to the crosslinking reaction and is proportional to the extent of reaction. According to the rheometry curve (rheography like Figure 12), the level of cure can be calculated from different torque values. There are three main time regions in a rubber vulcanization curve. The first regime is the induction period (*t*_s_) which provides a safe processing time. The second period is associated with the curing reaction, in which the network structures of rubber are formed increasing the stiffness. The network structures can include crosslinks, cycles, main chain modification, isomerization, etc. In the third period, the crosslinking network in the rubber is mature and some overcuring (reversion, equilibrium or additional but slower crosslinking) may occur depending on the rubber formulation and curing conditions. Overall, a curing curve exhibits a number of features which are applied to quantitatively compare the curing conditions. The main parameters are:

- *M_i_*: the initial torque indicates the stiffness (or shear modulus) of the unvulcanized rubber.
- *M_u_*: the ultimate torque indicates the stiffness of the fully vulcanized rubber which is related to the crosslink density of the rubber.
- Δ*M* = *M_u_* − *M_i_*: the difference between the ultimate and initial torque.
- *M*_90_: the torque to achieve 90% cure which is calculated as [224]:
  (23) $$M_{90}=M_{i}+0.9\Delta M$$
- *t_s_*: the time to reach 5% of M90 (scorch time).
- *t_90_*: the optimum cure time, which is defined as the time to reach 90% of curing.
- *CRI*: the cure rate index or the slope of the curing curve which is calculated as [225]:
  (24) $$CRI\left(\%\;min^{-1}\right)=\frac{100}{t_{90}-t_{s}}$$

The quality of the resulting rubber compound is controlled to a great extent by a careful selection of the curing parameters, such as temperature and time. Therefore, it is necessary to understand the effect of each curing parameter on the final foam properties.

#### 6.3.1. Curing Temperature

**Figure 13:**
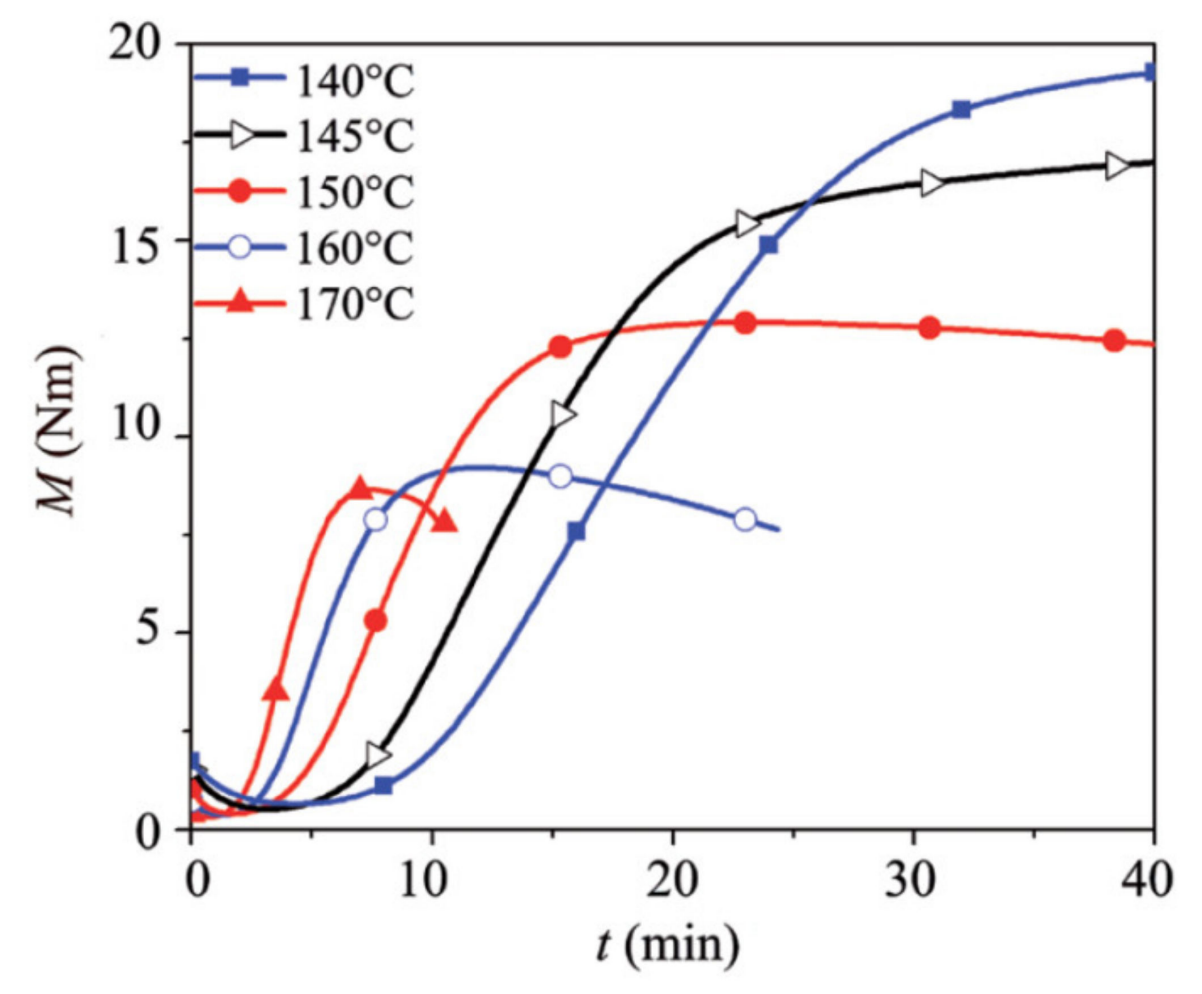
Curing behavior of IR foams at different temperatures [169].

As mentioned above, the curing characteristics highly depend on temperature and a great deal of literature reported the effect of curing temperature on vulcanization. Zakaria et al. found that increasing the temperature resulted in lower *M_i_* and *M_u_* [199]. This was related to crosslink density decreasing with increasing processing temperature and because homogenous mastication at high temperature can reduce the rubber compound viscosity. Similar results were reported by Vahidifar et al. stating that higher temperature improved chain mobility (lower flow resistance = lower viscosity) leading to lower *M_i_*, while increasing the temperature generated and released a higher volume of gas decreasing *M_u_*, and thus Δ*M* (Figure 13) [169]. Pechurai and coworkers reported that increasing the processing temperature resulted in lower scorch and cure time due to faster curing reactions [83]. It should be noted that the optimum curing temperature is not necessarily the same with or without CFA because thermal decomposition has a direct effect on the amount of heat generated (exothermic) of consumed (endothermic), thus affecting the curing characteristics.

#### 6.3.2. Precuring Process

**Figure 14:**
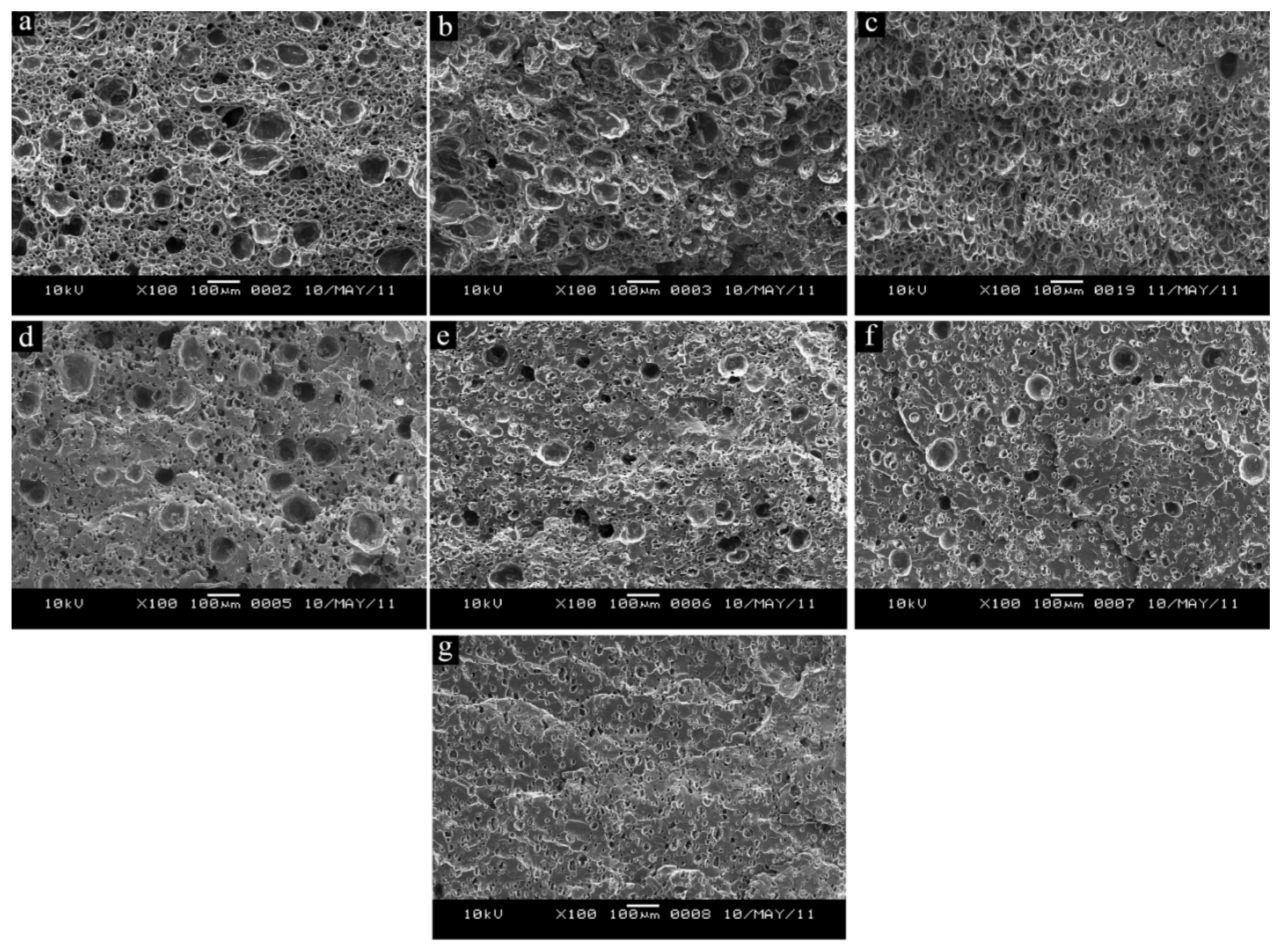
SEM images of BR/SBR/NR foams with various precuring levels: (**a**) 0%, (**b**) 20%, (**c**) 30%, (**d**) 40%, (**e**) 50%, (**f**) 60% and (**g**) 70% [226].

A study on BR/SBR/NR foams was reported by Wang and coworkers [226]. They produced blend foams via a two-stage compression molding process with different precuring levels from 0% to 70% and investigated the effect of precuring conditions on the final foam properties. The results indicated that the number of cells was higher at a precured level of 30% compared to the other level investigated. With increasing precured level, the average cell size decreased, thicker cell wall was produced, and the cell size distribution became narrower as shown in Figure 14. In addition, the initial torque, ultimate torque, curing rate and foam density increased with increasing level of precuring.

**Figure 15:**
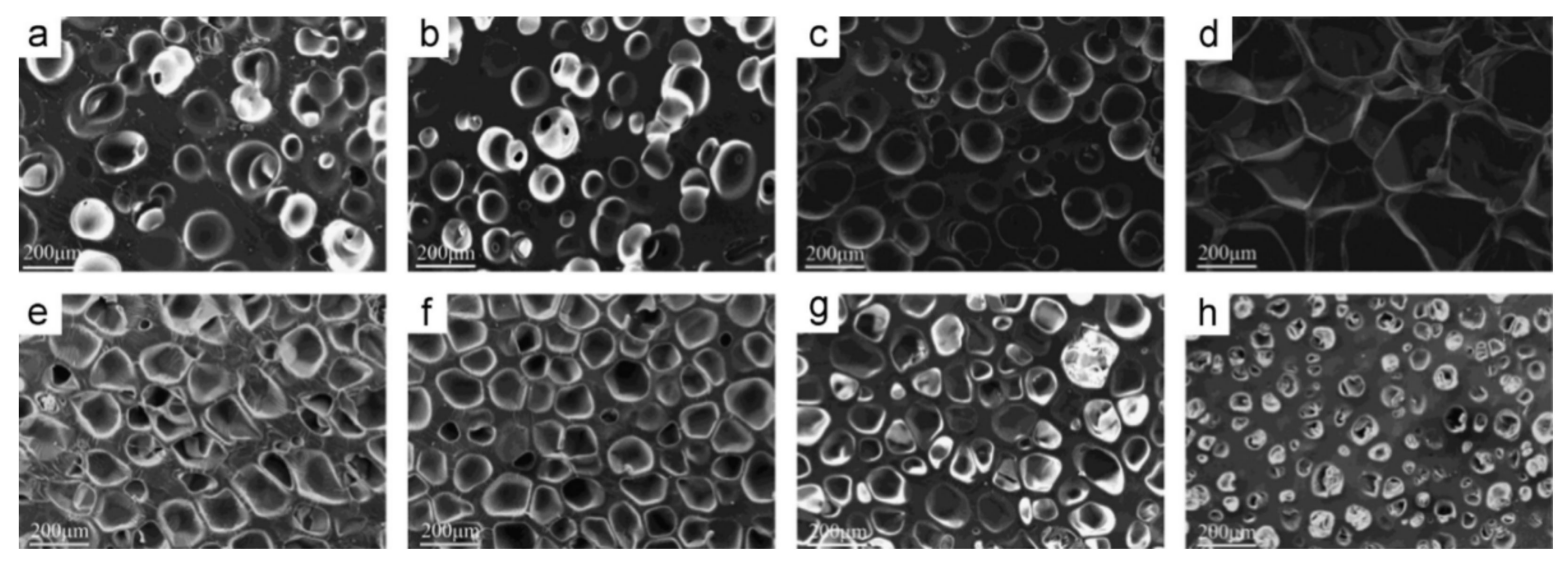
SEM micrographs of silicone rubber foams prepared under different precuring times. The silicone rubber was saturated at 10 MPa for: (**a**) 0 min, (**b**) 2 min, (**c**) 4 min, (**d**) 5 min, (**e**) 6 min, (**f**) 7 min, (**g**) 8 min and (**h**) 10 min [228].

As seen in Figure 14, large cells have been produced without precuring. However, as the precuring level increases, the number of large cells decreased down to a point where they disappeared at 70% of precuring. In addition, the overall cell size decreased with increasing amount of precuring. The optimum precuring level of 30% was explained by a competition between higher cell wall stiffness limiting cell expansion, but also cell wall rupture (coalescence) and at low viscosity the chains reorganized and the bubbles grow, which led to pressure decrease and the bubble being stable. Furthermore, the mechanical properties showed that increasing the precuring degree resulted in higher tensile modulus, tear strength, tensile strength, and elongation at break. The main reasons were associated to higher foam density and crosslink density, as well as thicker cell wall with increasing precuring degree. Phiri et al. compared the effect of precuring (P-C) and free foaming (F-F) on the morphology of reclaimed tire rubber foam composites [227]. Precured samples had fewer microcells than the free foaming sample. It was postulated that during the precuring stage, the foaming agent partially decomposed to release gas molecules and the foaming process started. Since at this step no significant curing occurs, the walls of small microcells tend to rupture easily to form larger cells. Song and coworkers studied the effect of precuring time on the density and pore morphology of silicone rubber foams [228]. Their results revealed that the crosslinked network structure was affected by the precuring time. In their study, 5 min was selected as the optimum precuring time to achieve the lowest foam density and the most uniform pore microstructure. Figure 15 presents SEM micrographs of the silicone rubber foams at various precuring times. From 0 to 4 min, spherical pores are distributed separately in the matrix. At 5 min, polygonal pores are opened and interconnected, while polygonal pores not connected with each other were obtained between 6 and 8 min. For 10 min of precuring time, the pores are very small. In fact, the strength of uncrosslinked or slightly crosslinked silicone rubber foams (0–4 min precuring time) was so weak that the bubbles are easily combined, or gas escaped from the rubber. For intermediate level of crosslinking (5 min precuring time) the silicon rubber toughened by the crosslinked network had limited flexibility hindering the gas molecules from escaping the matrix. However, highly crosslinked rubbers (6–10 min precuring time) had more strength and the free volume between the rubber molecules was smaller. Consequently, the gas molecules could hardly enter the rubbers and the rubbers generated an elastic restoring force, preventing the gas molecules to enter the matrix during foaming.

#### 6.3.3. Crosslink Density (CLD)

Elastomers are generally crosslinked in a random manner. Rubber crosslinking leads to substantial increase in elastic modulus, a marked increase in hardness and usually a reduction in the ultimate elongation and permanent set [229]. Crosslinking in rubbers mostly refers to covalent bonds linking the rubber’s chains together. These chemical bonds are mainly based on sulfur, and are composed of monosulfide (–S–), disulfide (–S–S–) and polysulfide (–S_x_– with x ≥ 3). Because there is a direct relation between CLD and mechanical properties, controlling the crosslink density is a key factor to determine the final properties of a product [230]. 

Two integrated approaches of the equilibrium swelling theory and thiol-amine analysis have been adapted to estimate and chemically analyze the crosslinks in cured rubbers immersed in solvents [231,232]. For this purpose, firstly the thiol-amine reaction led to selective chain cleavage of the sulfuric bonds. Secondly, the rubber-filler interaction theory (Kraus equation) coupled with the equilibrium swelling theory (Flory–Rehner equation) were applied to quantitatively determine the CLD by entropy changes due to polymer-solvent mixing.

(25) $$v_{cross}\left(mol.g^{-1}\right)=\frac{1}{2M_{c}}=-\frac{ln\left(1-v_{r}\right)+v_{r}+\chi v_{r}^{2}}{2\rho_{r}V_{s}\left(\sqrt[{3}]{v_{r}}-\frac{v_{r}}{2}\right)}$$

(26) $$v_{r}=\frac{\frac{W_{before}-W_{filler}}{\rho_{r}}}{\frac{W_{before}-W_{filler}}{\rho_{r}}+\frac{W_{after}-W_{before}}{\rho_{s}}}$$

(27) $$\chi =\beta +\frac{V_{s}}{RT}{\left(\sigma_{p}-\sigma_{s}\right)}^{2}$$

where *M_c_* is the average molecular weight of the rubber between the crosslinks, *V_r_* is the volume fraction of the equilibrium swollen rubber, *χ* is the Flory–Huggins polymer-solvent interaction parameter, vs. (cm^3^ mol^−1^) represents the molar volume of the solvent used and *ρ_r_* (g cm^−3^) is the density of rubber [233]. In Equation (26), *W_before_* (g) and *W_after_* (g) describe the weights of the rubber sample before and after swelling respectively, while *W_filler_* (g) is the weight of the filler and *ρ_s_* (g cm^−3^) is the density of the solvent. In Equation (27), *β* represents the lattice constant for the polymer-solvent blends (*β* = 0.34), *R* is the universal gas constant, *T* (K) is the absolute temperature, *σ_p_* (MPa^1/2^) and *σ_s_* (MPa^1/2^) are the solubility parameters of the rubber sample (16.7 MPa^1/2^ for NR) and solvent (18.0 MPa^1/2^ for toluene) [234]. The Kraus equation is applied to distinguish between the chemical crosslinking density by the crosslinking agent and the rubber-filler interaction by the reinforcement agent [235,236].

(28) $$\frac{v_{r}-f}{v_{r}}=1-m\left(\frac{\varphi }{1-\varphi }\right)$$

By linear regression analysis on the filler concentration, Equation (28) can be obtained where (*v_r_* − *f*) and *v_r_* represent the volume fraction of the equilibrium swollen rubber without and with the filler respectively, *φ* is the volume fraction of the filler and *m* is the Kraus interaction parameter between the filler and the rubber. In addition, there are several other methods to analyze the structure of crosslink bonds, such as the Mooney–Rivlin model and nuclear magnetic resonance (NMR) [237,238,239]. 

With increasing the curing time, the amount of curing agent decomposition continuously increases resulting in a denser crosslinked network and a higher crosslinking density. At a low vulcanization degree, less crosslink chains can be formed, and the network structure would be vulnerable to destruction. When the vulcanization degree is sufficiently high, the number of crosslinking chains substantially increases, while the number of free chains strongly decreases. The length of crosslink chains and dangling chains also become shorter, and thus the interaction between molecular chains is enhanced [240]. It is known that the torque difference (Δ*M*) can be indirectly related to the crosslink density [241]. According to Shao et al. [96], *M_u_* − *M_i_* for SBR foams increased with increasing DCP content as a peroxide crosslinker.

In general, the incorporation of nanoparticles into the compounds increases the crosslink density of the foams. Bashir et al. attributed this to higher *M_u_* value while processing (higher stresses) [164]. The particles can also act as a starting point to improve the crosslinking between the main rubber chains and the crosslinker resulting at higher *M_u_* [242]. The crosslinking density during vulcanization is related to the level of curing limiting molecular mobility (flowability) [40]. The crosslinking density of NR latex foam reinforced with nanofibrillated cellulose (NC) increased with increasing NC content. This was associated to higher interaction between NC and NR, as well as hydrogen bonding between them [243]. The literature reported that higher foaming temperature resulted in lower crosslink density [212]. This trend can be associated to a breakdown of crosslinks under high stress as they swell [86].

## 7. Properties of Rubber Foams

### 7.1. Mechanical Properties

Although tensile tests are common for other materials, they are rarely used for foams. This is because of the difficulty of gripping foams to apply tensile loads, and partly due to the few applications involving higher tensile loads as most of the foams are weak in tension and fracture easily. In contrast, compressive loading is more common for applications like cushioning and packaging under different weights.

The mechanical behavior of foams depends on foam morphology in general, and the cell wall thickness in particular [244]. Increasing the dispersion of cell wall thickness results in lower Young’s modulus and shear modulus [245]. The results of Grenestedt et al. showed that the bulk modulus and shear modulus of Kelvin closed cell foams were reduced by 19% when the thickest cell walls are 19 times thicker than the thinnest one [246]. Ramasamy et al. studied the effect of rice husk powder (RHP) on the mechanical properties of NR foams [247]. According to their results, the tensile strength initially reduced at lower RHP content but then increased at higher amount of RHP. When the RHP content was low, the filler was not enough to reinforce the NR foam and RHP-matrix interaction was weak. The fillers also act as stress concentration points. Once the RHP concentration increased, higher adhesion and compatibility occurred between the RHP and the NR compound leading to higher tensile strength. Moreover, the elongation at break and modulus at 100% elongation results showed a decrease followed by an increase with higher RHP content. The aggregation tendency of RHP particles inside the matrix restricted the mobility of flexible rubber chains. Hence, the foam elasticity decreased, while the stiffness and rigidity increased [248]. In another study, it was concluded that the surface of the filler played an important role on the mechanical properties improvement of NR foams [249]. In this regards, smaller kenaf particle sizes inside NR increased the foams tensile strength, modulus at 100% elongation, compression strength, compression set and hardness. The main reason was that the smaller filler size provided more contact area for rubber chains to interact and forming physical crosslinking resulting in less mobility of the rubber chains and foam higher stiffness. Sritapunya investigated the compression of NR foams using various amount of synthesized salt based foaming agent [250]. This foaming agent was synthesized by purging the excess of CO_2_ into a mixture of amino alcohol, methanol, and water until all of the liquid was changed into a solid (salt). The thermal reaction of this salt led to CO_2_ gas and vapor release including amino alcohol that is an environmentally friendly agent. The results showed that a foam with 1 phr of salt had the highest compressive stress compared to that of foam rubber using 0.4 phr of commercial foaming agent. This phenomenon was attributed to two reasons. Firstly, the foam with 1 phr of foaming agent formed larger cells compared to the foam based on the commercial foaming agent. These large cells contained higher amount of gas, which could well relieve the stress from compression. The second reason was that the higher gas escaping in the higher foaming agent content foam are imposing stresses on the rubber chains and improving molecular orientation. Therefore, this orientation led to strength enhancement of rubber foams. Yamsaengsung et al. reported that the tensile modulus for prepared EPDM foams, using two CFA types (OBSH and EPR-b-OBSH), decreased with increasing foaming agent content [251]. This was due to a decrease of the load bearing area in the solid EPDM by the formation of the gas phase. The tensile modulus of EPDM foams with OBSH was greater than that with EPR-*b*-OBSH due to a higher number of cells. Furthermore, higher foaming agent content resulted in higher compression set for both EPDM foams. In these cases, the recoverability of the EPDM foams decreased with increasing both types of foaming agents. This effect was more important for EPR-*b*-OBSH. This can be attributed to the deformation of the cellular structure after compression leading to a loss of elastic recovery from the gas phases. In this case, the compression set for EPR-*b*-OBSH foamed EPDM was higher than the value of OBSH ones because the latter had higher cell density.

### 7.2. Acoustic Properties

**Figure 16:**
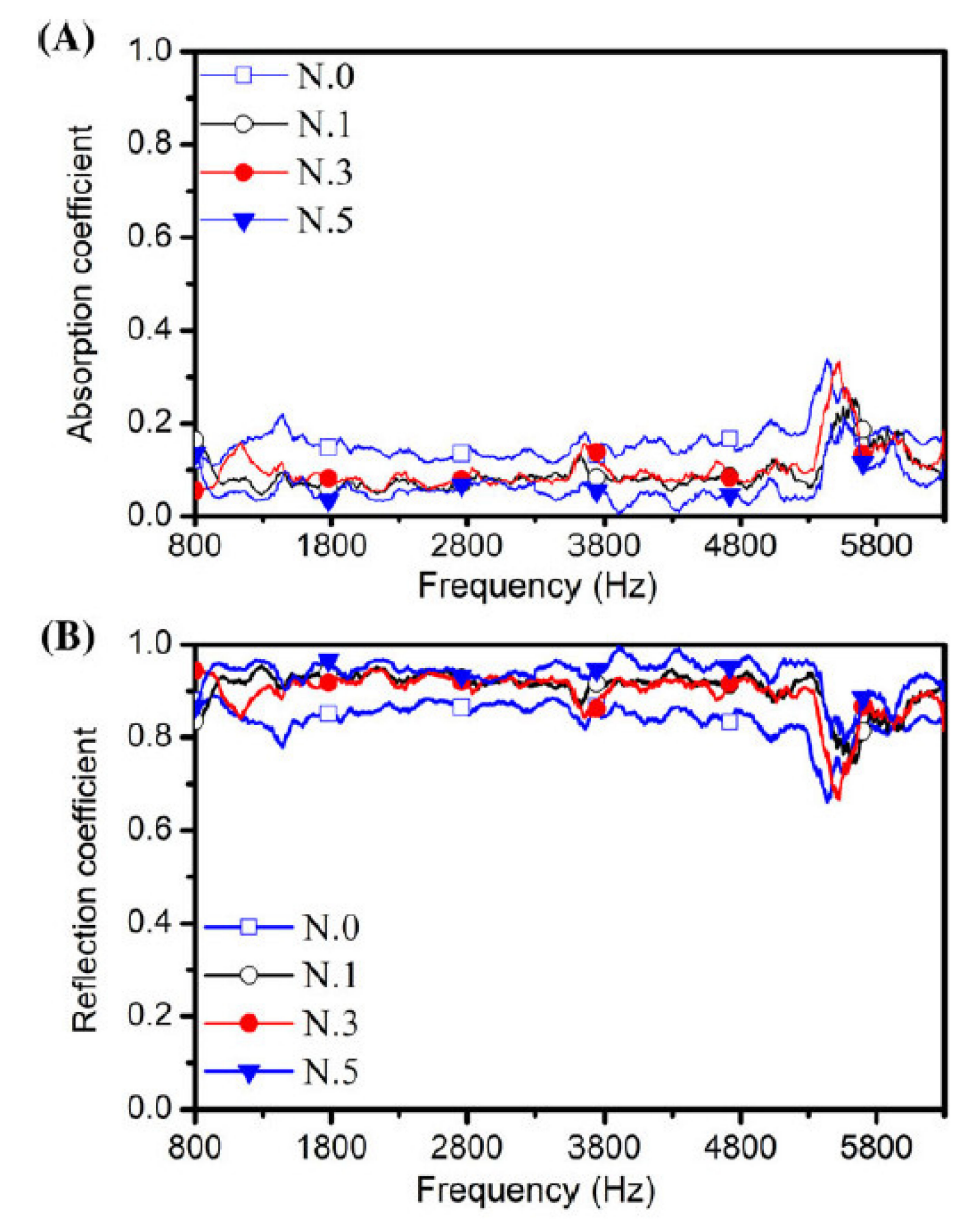
Sound absorption coefficient (**A**) and sound reflection coefficient (**B**) as a function of frequency for NR/NC/NCB hybrid nanocomposite foams with different NC content [161].

One of the biggest problems associated to human and environment health is noise pollution [252]. Rubber foams, as a lightweight and low-cost material, have addressed this issue due to their vibration control and noise absorption wave’s abilities in a wide frequency range [253]. The literature showed that open cell foams are more suitable for sound insulation applications compared to closed cell foams. Sound absorption and sound insulation are the main acoustic properties in engineering applications [254]. The sound absorption properties are different from transmission loss to insulation leading to vibration decreasing over time. The origin of sound absorption is either due to friction between two phases as a result of internal friction or due to hysteresis of the material [255]. In general, two mechanisms of viscous losses and thermal losses are involved in sound absorption. The former originates from the friction between the air molecules and the matrix, while the latter is related to the heat transfer inside the solid phase [256,257]. Once the sound waves comes into the foam, there are large amounts of sound energy losses with low amount of reflection. In other words, the sound waves hitting the rubber surface generates pressure form the sound waves leading to deformation inside and outside the cells. These flows convert the energy of sound waves into heat. In this regards, Najib and coworkers investigated the effect of foaming temperature on the acoustic properties of NR foams [77]. The expanded foam at 140 °C indicated great elastic behavior and a superior sound absorption coefficient. This was attributed to the fine cell characteristic; i.e., smaller average cell size, thicker cell walls and higher cell density, obtained at this temperature. It was concluded that the sound absorption capability of NR foams was related to the stiffness of the solid portion; i.e., it is controlled by the viscoelastic properties of the base material. Vahidifar et al. reported that the effect of NC content and foam morphology on sound absorption and/or reflection was negligible (Figure 16) [161]. They stated that the remarkable impedance difference between NR matrix and air led to very low penetration of sound waves and thus more sound reflection at the air–rubber interface. They also reported that the sound absorption coefficient of NR foams decreased with increasing CB content [46]. They justified this with two reasons. Firstly, a softer foam material absorbs sound more via the vibration of the matrix. Hence, increasing the CB content led to more softening and thus reduced sound absorption coefficient. Secondly, the foams with smaller cell size exhibit low sound absorption. On the other side, increasing the amount of CB resulted in lower cell size for NR foam leading to lower sound absorption. Zhang et al. showed that the incorporation of mechanochemically devulcanized GTR into PU foams had excellent acoustic absorption properties in the middle-frequency region [258]. The addition of mechanochemically devulcanized GTR particles into the PU matrix increased the viscoelastic properties and improved the mobility of rubber chains. When the sound wave struck the PU/GTR foam, the GTR particles underwent a highly elastic deformation due to the movement of rubber molecular chains, which was characterized by a significant lag between the deformation and the stress.

### 7.3. Thermal Insulation Properties

The excessive consumption of fossil fuels has led to a number of ecological and environmental problems, such as environmental pollution, global warming and ecological deterioration in the world [259,260]. Polymeric foams are thermal insulation materials with low thermal conductivity playing an important role in increasing the energy efficiency in fields like packaging, construction, transportation and aerospace industries [261]. To study thermal insulation materials, the effective thermal conductivity is an important heat transfer property of the materials [262]. As is well-known, lower thermal conductivity leads to better insulation ability [55]. Bird feathers are a natural example of a thermal insulation containing hollow cells made of keratin fibers. 

Thermal conductivity is a material property describing its ability to conduct heat and is reported in W/(m·K). Thermal conductivity is mainly function of the cell morphology, cell size and foam density. Several results showed that closed cell foams are more appropriate than open cell foams for thermal insulation purposes [263]. In fact, the separated cells in the closed cell foams can trap the low amount of thermal conductive gases. It was shown that polymeric foams with several small cells have less heat transfer than foams with a smaller amount of larger cells [208]. Gao et al. concluded that the thermal insulation properties of hollow glass bead (HGB) filled SR foams were related to the amount of foam porosity [264]. They revealed that the thermal conductivity increased with higher HGB content and larger particle size because both factors led to a lower porosity of the materials. Similar results for the effect of filler size on the thermal conductivity of EVA/NR foams were examined [265]. They indicated that the EVA/NR foam with nanoclays had better beneficial effect on the thermal insulation over china clay because these nanoclay-based foams contained a relatively large number of small cells due to the small size of these nanoparticles. Charoeythornkhajhornchai et al. reported that the high amount of insulating gas bubbles in the NR matrix resulted in low thermal conductivity because gas bubbles have lower thermal conductivity than NR [154]. Jia and coworkers prepared microcellular SR foam without surface defects [262]. The thermal conductivity of their foams obtained a minimum value of 0.14 W/m·K, and the thermal insulation was improved by 39% compared with SR composites.

### 7.4. Thermal Degradation Stability

**Figure 17:**
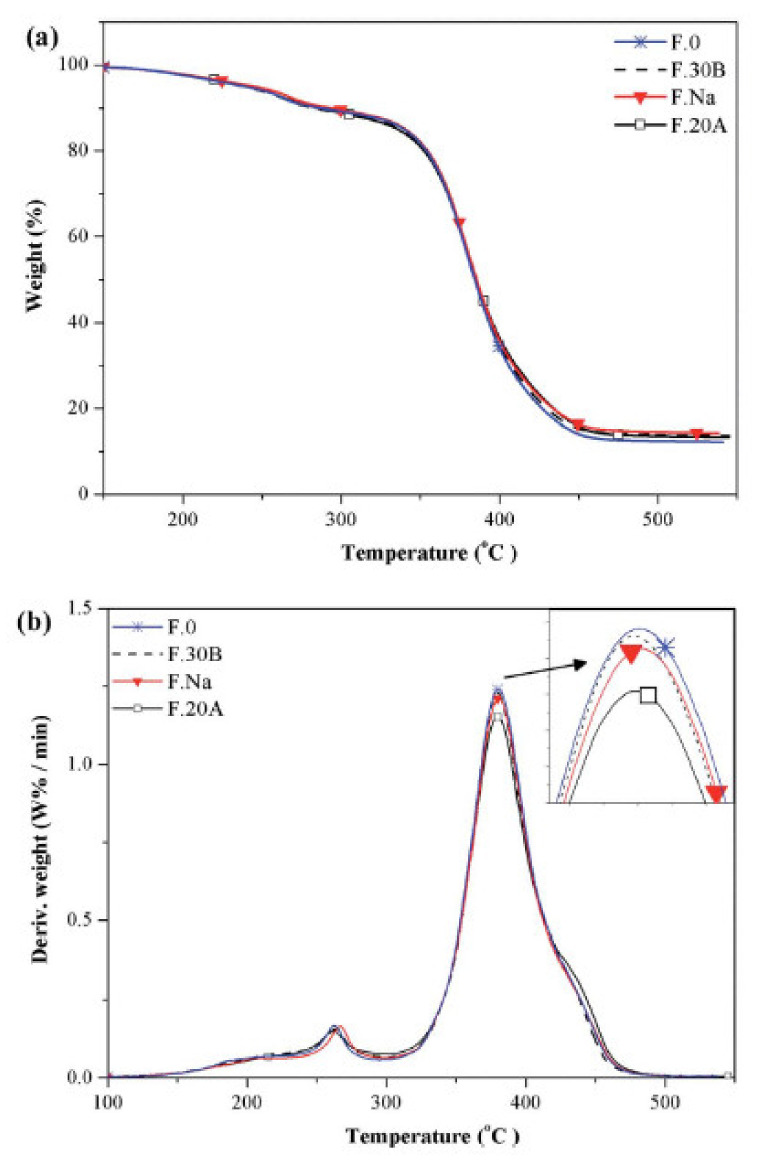
Thermal degradation behavior of NR/NC foams: (**a**) TGA and (**b**) DTG [52].

Thermal degradation (stability) is a very important property of rubber foams to maintain their required properties, such as strength, toughness or elasticity, according to the temperature they are subjected to [266]. A detailed understanding of how rubber foams break down upon heating is important in the design of materials with improved properties for a particular application. TGA, DTA and DSC are mainly used to quantify the thermal stability of rubber foams. TGA provides a direct measure by observing the temperature(s) at which actual weight loss occurs. DTA/DSC can be used to determine the safe storage and use temperature of materials decomposing exothermally by finding the temperature at which these decompositions occur. Shao and coworkers found that the crosslink density plays an important role in the thermal stability behavior of SBR foams [96]. They observed that the thermal decomposition temperature increased by applying two various crosslink agents simultaneously. It was attributed to higher crosslink density obtained by a double crosslinker system. Different works reported a significant thermal stability improvement of SR foams by the addition of fillers including nanographite, carbon nanotubes and carbon black [201,262,267]. This was related to a physical barrier effect caused by the addition of fillers in the rubber matrix acting as blocking layers and increasing the tortuosity hindering the diffusion and emission of the volatile decomposition products. Figure 17 presents typical TGA and DTG curves for NR/NC foams with different NC types in an inert atmosphere [52]. The thermal degradation behavior of all the samples exhibited two-step decomposition. The first step was related to small molecules and additives such as oil, curing system, foaming agent and so on. The second decomposition step is attributed to the NR chains degradation. The addition of NC had limited effect on the thermal stability properties. In this case, the NC’s barrier effect was negligible because the cell wall was too thin and the tortuosity pathway made by the nanofiller was not important.

### 7.5. Electrical Properties

Since all polymers have electrical insulating properties, various electrically conductive fillers such as CB, exfoliated graphite, CNF or CNT are added to convert them to composites for antistatic and EMI shielding applications [268,269,270,271,272,273,274]. However, the presence of these fillers alone cannot induce electrical properties, and their uniform dispersion inside polymer matrices is one of the most promising methods to reduce the percolation threshold [275]. This critical filler content is related to a several orders of magnitude variation in electrical conductivity due to the formation of continuous electron paths or conducting networks [276]. The amount of conducting filler must be above the percolation threshold to achieve conducting networks in the composites. Fillers much longer than they are wide (high aspect ratio) are more likely to contact each other and thus lower electrical resistivity and higher EMI shielding is produced [277]. For instance, CNT are very high-cost fillers, but have high aspect ratios and low percolation threshold (<5 wt.%) to enhance conductivity compared to CB [278]. Nevertheless, there are few studies on the electrical properties of rubber foams. Fletcher and coworkers designed elastomer nanocomposite foams based on FKM/MWCNT [279]. The percolation threshold was obtained at 2% CNT and the saturation conductivity occurred at 8 wt.% Combining the good electrical properties with the flexibility and fluid resistance of fluorocarbon yields a very versatile yet lightweight material for EMI applications. El-Lawindy and coworkers investigated the electrical behavior of reinforced EPDM and NBR foams with different ADC concentrations [280,281]. From DC conductivity measurements, it was reported that increasing the ADC content substantially modified all the electrical parameters. A competition between two conduction mechanisms was also observed: tunneling and thermal activation.

## 8. Applications of Rubber Foams

### 8.1. Energy Absorption Applications

**Figure 18:**
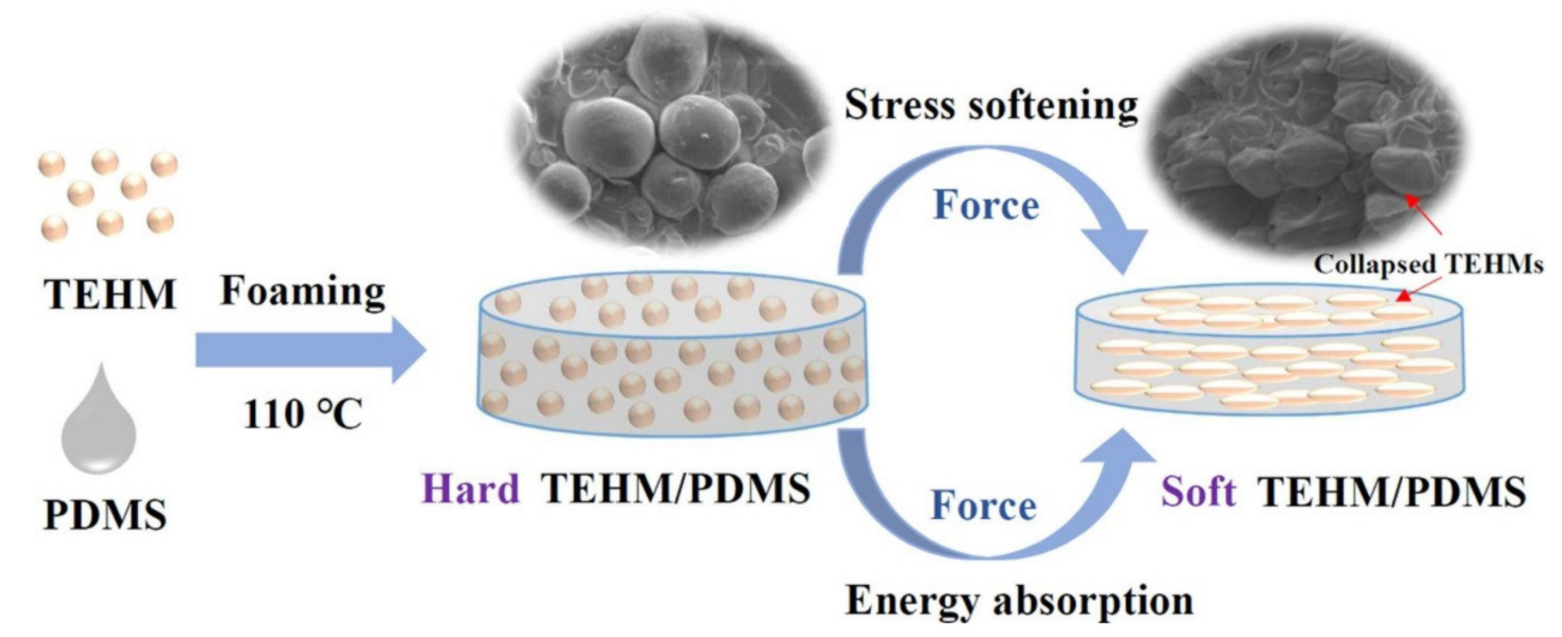
Schematic representation of PDMS/TEHM foams preparation with improved softening performance and high motion-energy absorption efficiency [289].

Rubber foams have elastic properties making them applicable as shock and vibration dampers [282,283,284]. They can dissipate and/or undergo impact energy over a range of frequencies for specific applications, especially under cyclic deformation [285,286]. Energy-absorbing polymeric foams are widely used in the automotive industry to mitigate impact stresses and prevent injuries to the occupants in the event of collisions. Sanborn and coworkers applied a modified Kolsky compression bar setup to evaluate the frequency domain energy dissipation performance of silicone foams [287]. They were able to fabricate silicone foams with 99% dissipation of impact energy over a frequency range of 1–8 kHz. In another work, their group claimed that increasing the impact speed to 12.8 m/s, the dissipation of the impact energy at small prestrains by silicon foams could be close to 100%. On the other hand, higher increase of prestrain decreased the energy dissipation ratio to 59% [288]. Cai et al. prepared PDMS foams using different weight ratio of thermo-expandable hollow microspheres (TEHM) as shown in Figure 18 [289]. Expanding the TEHM in the PDMS matrix led to foams with improved mechanical properties and unique softening performance. This softening behavior provided the foams with great toughness and high motion-energy absorption efficiency. For instance, the TEHM/PDMS (1:2) foams exhibited an energy absorption of 0.8 MJ/m^3^ and superior *I* value (ideal energy absorption efficiency) of 74%.

### 8.2. Pressure Sensor Applications

**Figure 19:**
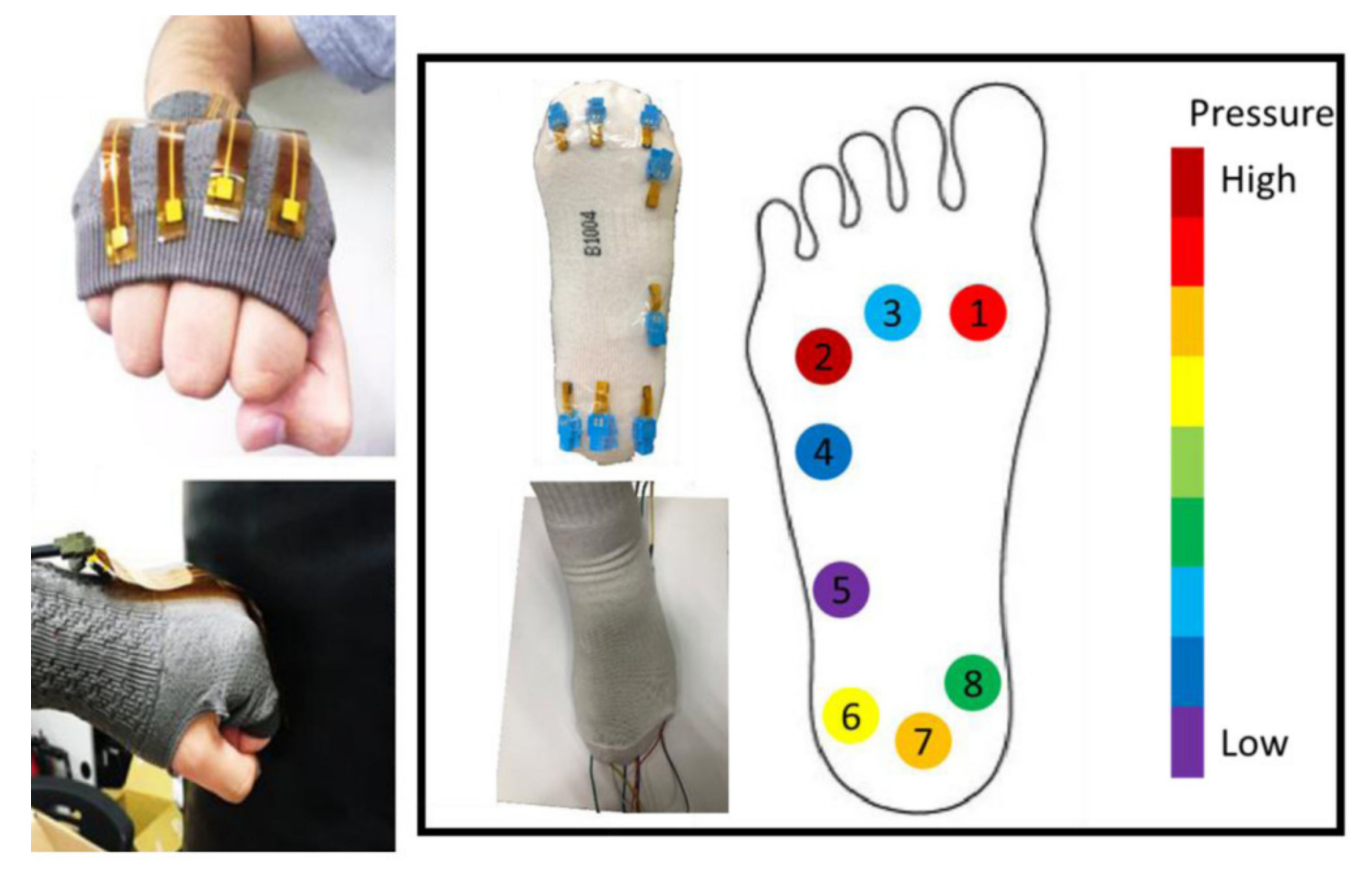
Pressure sensor application of fluorine rubber and CNT foam [295].

Flexible pressure sensor foams as fundamental parts in wearable electronic devices which are extensively studied due to their low cost, simple working mechanism, easy signal processing, simple manufacturing process and high sensitivity detection over a wide pressure and deformation range [290,291,292,293,294]. These piezoresistive materials can transduce external pressure into internal resistance variation being detectable in the form of current. Hsiao et al. developed a simple and effective method to prepare porous conductive nanocomposite with high stability for pressure sensing applications (Figure 19) [295]. A solid PSR is firstly prepared by mixing fluorine rubber and CNT showing electric resistance variation to compressive stresses with a sensitivity of 0.35 MPa^−1^. To enhance this sensitivity, a foaming agent (DPT) was mixed in the PSR to create porous structures with controllable pore sizes. With 18 phr DPT, the porous PSR exhibited an optimum sensitivity of 4.31 MPa^−1^ due to higher deformability. Zhang and coworkers reported an ultrafast, convenient, scalable and cost-effective strategy to fabricate flexible strain sensors based on NR/graphene foams [296]. Due to the unique macrostructure of graphene, these strain sensors exhibited a good trade-off between opposing properties such as sensitivity and stretchability to measure multiscale motions. The gauge factor of the flexible sensor based on this method reached 210 in the strain range of 10–40%. It also showed a good compromise between sensitivity and compression performance to monitor different human activities, such as movement steps, finger bending and identifying different strains associated to human body motion.

### 8.3. Bioapplications

**Figure 20:**
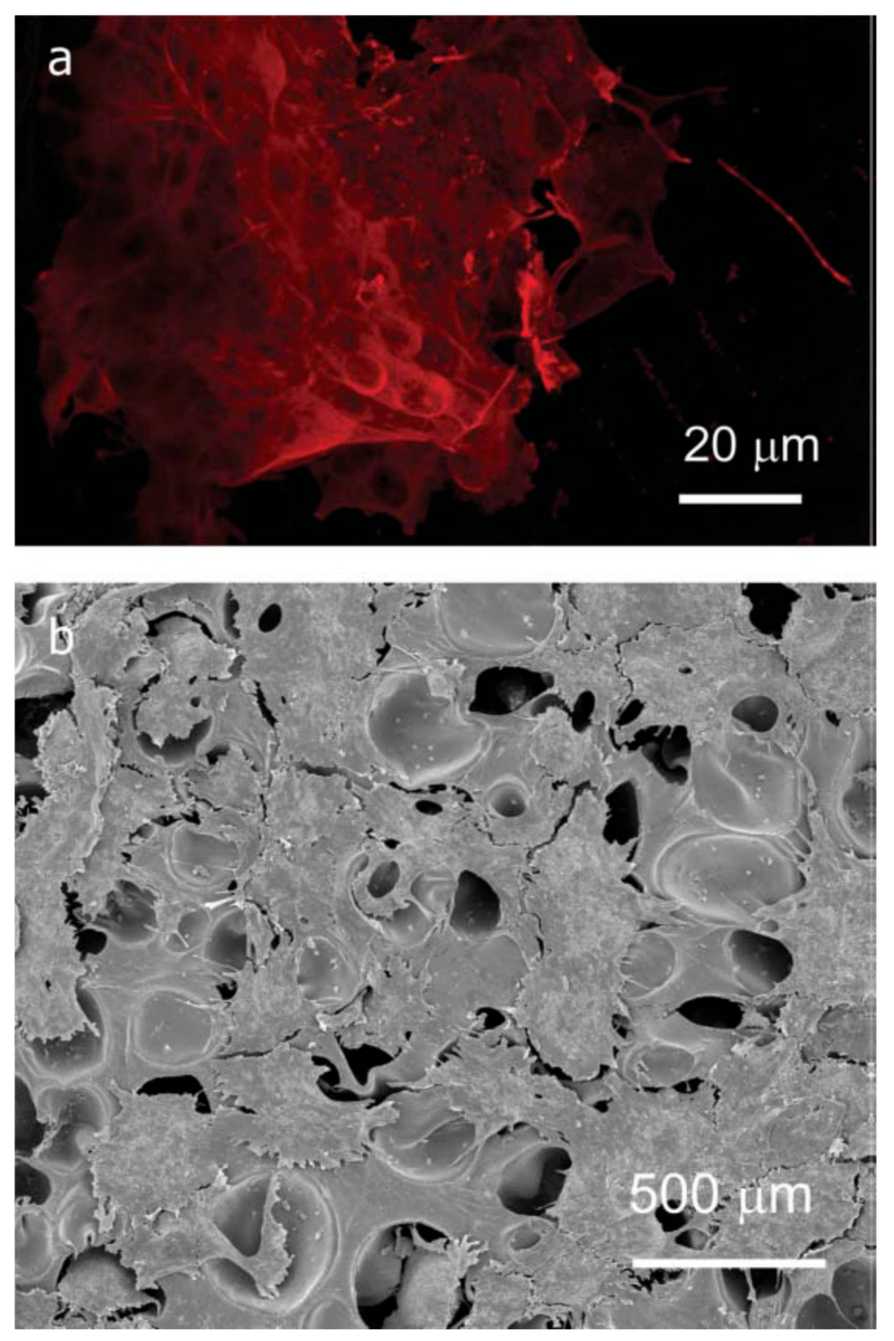
(**a**) Fluorescent microscopy image of neuroblastoma (human neuron-like, SH-SY5Y) cells cultured on foamed 30/70 SIS/THFMA blends. Note the intense red staining of the neuronal cytoskeletal protein b-III tubulin in the cluster of cells shown here. (**b**) Electron micrograph of ovine meniscal chondrocytes cultured on foamed 30/70 SIS/THFMA blends [297].

Despite the biocompatibility properties of some rubber foams, they are rarely used in biomedical applications. Barry et al. reported the use of scCO_2_ in the foaming of styrene-isoprene-styrene/tetrahydrofurfuryl methacrylate (SIS/THFMA) at three compositions as scaffolds for tissue engineering [297]. Higher THFMA contents resulted in higher swelling. The foam with 70% of THFMA, compared to those containing 30% and 50% THFMA, displayed higher open cell content in its whole structure and had larger cell size and cell size distribution. According to helium pycnometry results, the 30/70 SIS/THFMA sample showed the highest interconnectivity in its cellular structure. Figure 20 presents the cell culture experiments. Their results confirmed that the SIS/THFMA scaffolds are able to hold adhesion and growth of neuron-like cells and chondrocytes for more than 4–5 days. Chayaphan and coworkers designed NR composite foams using tourmaline powder as filler to improve the far infrared (FIR) emission to improve blood circulation [6]. The skin temperature increased by 1.6 °C with 300 ppm tourmaline which was the highest average skin temperature increased, while 1.5 °C, 1.1 °C and 0.6 °C were observed with 500 ppm, 100 ppm and without tourmaline, respectively. The skin temperature elevation related to blood circulation and FIR from tourmaline might is accelerating the blood circulation under a skin layer. The group of Rathnayake reported that the incorporation of alkaline solution of silver nanoparticles into NR latex foams can enhance the antibacterial activities of these foams [298]. Furthermore, the modified NR latex foam gave antifungal properties which were absent in the control NR foam samples. 

### 8.4. Absorbent Applications

**Figure 21:**
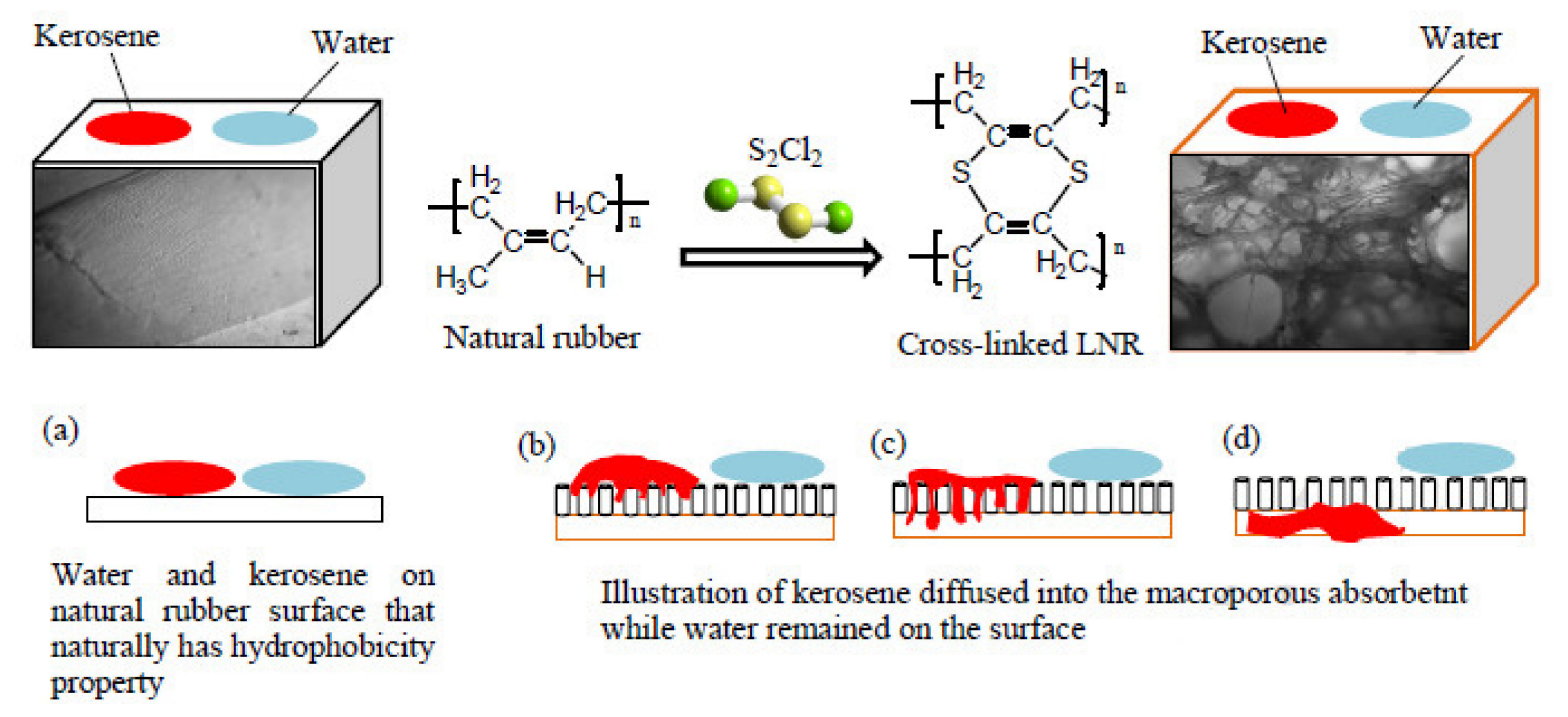
Absorption of oil (kerosene) and water molecules on different surface of: (**a**) NR and (**b**–**d**) the prepared absorbent foam [300].

Hassan and coworkers studied the gamma irradiation influence on the potential application of composites based on EPDM/clay foams for the removal of different types of dyes from aqueous solutions [299]. They used various types of clays as adsorbents for different classes of dyestuffs (basic, acid, reactive and disperse) from aqueous solutions. The Na-montmorillonite gave maximum adsorption affinity toward basic dye, while the Aswan clay gave maximum affinity toward acid dye. Both types of clay did not show any response toward reactive and dispersive dyes. The results showed that a radiation dose at 50 kGy was the optimum for dyes removal, while improving the soluble fraction and swelling ratio of EPDM rubber composites with clays. Lazim et al. designed a novel macroporous and hydrophobic absorbent foam from modified NR via vulcanization at different crosslinker (S_2_Cl_2_) concentrations (Figure 21) [300]. The crosslinking density showed that increasing the crosslinking agent content resulted in a more oleophilic absorbent material. The foams presented rapid absorption for several types of oil including high viscosity oils such as diesel, olive and hydraulic. Their prepared absorbents were able to absorb the oil up to 9.39 g/g which was comparable to other commercial absorbents like polypropylene mats and cotton fibers with 9–15 g/g and 6–11 g/g absorption, respectively. Riyajan and coworkers fabricated macrocellular foams based on maleated ENR/PVA blends for organic solvent/oil absorption properties [301]. They used various MC content as a crosslinker and foaming agent. The maximum moisture content and moisture absorption was found to be 2% *w*/*w* MC due to its highly porous structure. The blend foams revealed excellent absorption capabilities toward various oils.

The MC concentration had an obvious effect on the absorption level and the optimum MC concentration was 2% generating the highest adsorption capacities for some organic solvents. In another work, Panploo and coworkers developed an eco-friendly NR latex foam to adsorb CO_2_ gas under environmental temperature and pressure [302]. They used two different methods including overhead stirrer (OS) or cake mixer (CM) to produced NR latex foams. The prepared samples by CM were incorporated by either unmodified silica (CM-USi) or APTES-modified silica (CM-MSi) for improving the CO_2_ adsorption capacities. The CM foams had larger cell sizes than OS foams because of their higher capacity to adsorb CO_2_ gas. Adding USi or MSi particles into CM foams, the CO_2_ adsorption capacity was improved by over 2.6 and 2.87-fold respectively, compared with the unfilled CM foam. A superhydrophobic and superoleophilic EPDM rubber foam modified with methyltrichlorosilane (MTCS) with desirable oil/water selectivity was fabricated by Liu and coworkers [303]. Their manufactured rubber foams showed excellent repellence behavior towards water, while a significant absorption capacity of 8–12 times of their own weights for a wide range of viscosity (from 0.3 to 400 mPa.s) was observed.

## 9. Conclusions

### 9.1. General Conclusions

Rubber foams, as a type of lightweight material, have several superior properties, such as low density, high elasticity, great resistance to abrasion, good thermal and acoustic insulation, high specific strength and tensile strength which are widely used in civil and industrial applications, such as insulation, packaging, automobile, aircraft, medical and construction industries. The structure of polymer foams can be divided into open cell or closed cell groups with pores in the range of millimeter to nanometer depending on the processing method, formulation and foaming conditions form their synthesis. 

The rubber foaming process can be decomposed into three steps: nucleation, growth and stabilization. Firstly, nuclei are created as small discontinuities or cells in the rubber matrix, and then these cells grow in volume, while the curing process leads to a stabilization of this cellular structure. In the preparation of rubber foams, two important parameters can affect the properties of a final product. The first parameters are related to formulation and the second parameters are related to the processing conditions. The types of rubber, accelerators, fillers and foaming agents, as well as their amounts are related to the formulation category, while the foaming (temperature, pressure and one-stage or two-stage expansion) and curing (temperature, time and precuring time) steps are related to the processing itself. Therefore, the morphological, physical and mechanical properties of rubber foams are strongly dependent on these parameters. In this review, most of the researches done on the effect of these parameters on the final properties of rubber foams have been discussed. In addition, the formulation, foaming chemistry, curing, processing, foam morphology, properties and some applications of porous rubbers were discussed. It was shown that a wide range of properties can be obtained leading to applications in different fields. Nevertheless, more work is needed to completely understand the relations between formulation, processing, structures and properties.

### 9.2. Opening for Future Work

In the early years of foam production, most of the efforts mainly focused on how to fabricate these cellular structures. Later, with the advent of nanotechnology, the works shifted to the use of different nanofillers to determine their effects on the properties of polymeric foams. However, research on nanocomposite rubber foams is still incomplete and is not comparable with nanocomposite studies on thermoplastic foams. The effect of the size, shape, and surface chemistry of nanoparticles on the rubber foam structure and properties still needs further study. On the other hand, the literature is full of blends of thermoplastic foams with several types of thermoplastics being used [304,305,306,307,308,309]. However, few studies were found on the foaming of rubber blends or/and thermoplastic/rubber blended foams. 

Although the effect of various parameters, including pressure, temperature, time, and precuring time, are well known for many porous rubbers, the theory of rubber foaming is still relatively complex. Furthermore, due to simultaneous foaming and curing, accurate understanding of these processes and their interactions still remains unclear. Despite several attempts to modify the classical nucleation theory, no model can currently provide a precise description of cell nucleation in rubber foams. Most of the time, cell nucleation and cell growth are modeled separately in the literature, even though in the rubber both processes are fully integrated and can occur simultaneously. Studies in this area are required to deliver accurate and predictive models for better description and prediction of cell nucleation and growth.

## Figures and Tables

**Table 1:** Types, properties and molecular structures of some important rubbers [93,94,95,96,97,98].

Rubber	Properties	Molecular Structure
NR	Biocompatibility; high elasticity, tear and abrasion resistance; high resilience, damping behavior and tensile strength; good oxidation resistance; low compression set; low ozone resistance; poor chemical resistance and processing ability	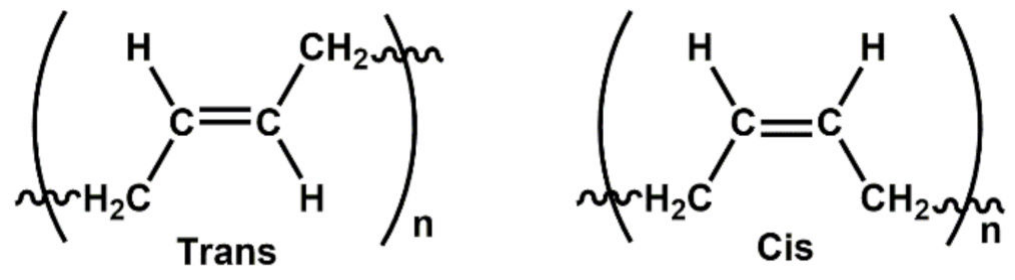
EPDM	High chemical stability; high tear, impact and abrasion resistance; high heat-aging (100 °C), weather, ozone and oxidation resistance; high resistance to break down	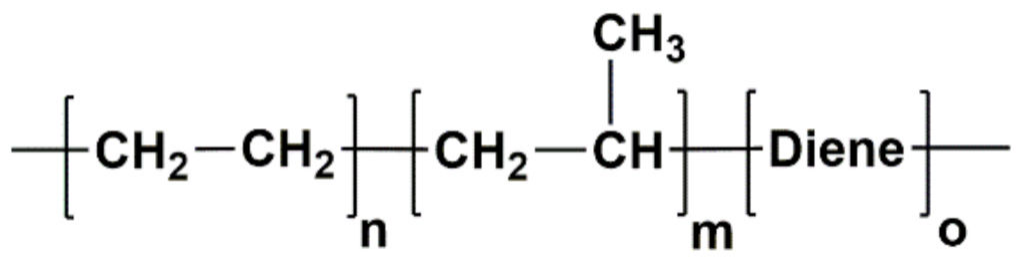
CR	Self-extinguishing behavior; good chemical stability; maintains flexibility over a wide temperature range	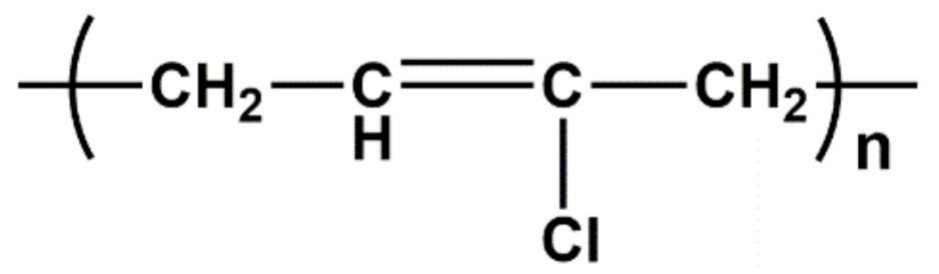
SBR	Good heat aging (100 °C); high elongation; good resistance to diluted and concentrated alkalis, alcohols and solvents	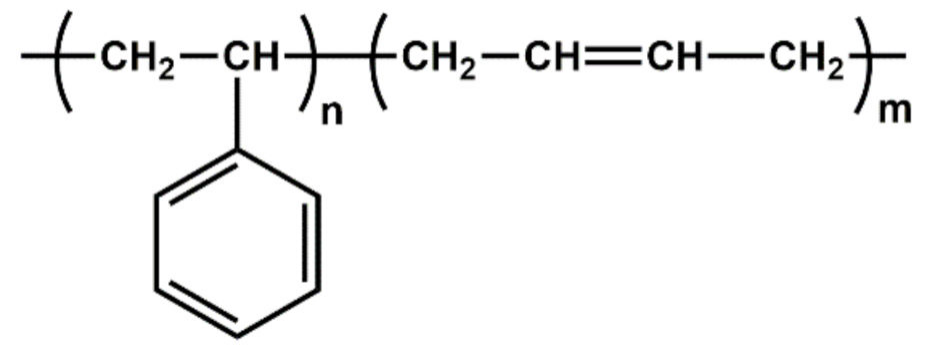
EVA	Low-temperature toughness; stress-crack resistance; hot-melt adhesive; waterproof properties; resistance to UV radiation	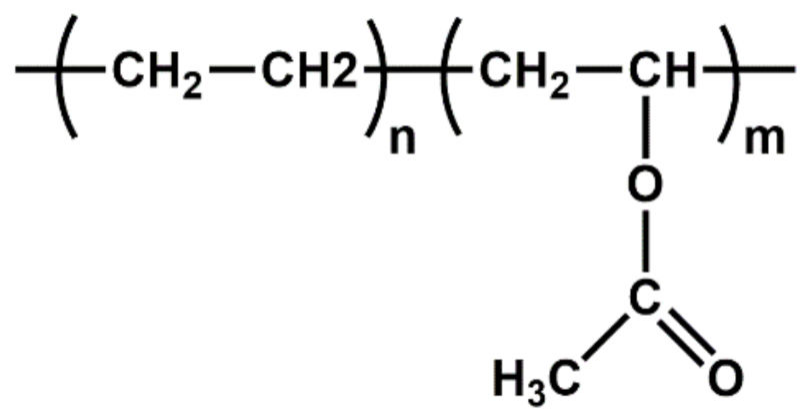
NBR	Resistance to nonpolar oils, silicon oils, fuels and ordinary diluted acids and alkalis; good oxidation resistance; good heat aging (100 °C); high thermal stability	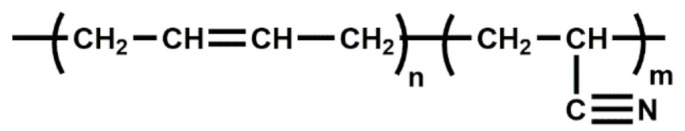
IIR	High resistance to heat, oxygen and ozone; low gas and moisture permeability; poor resilience	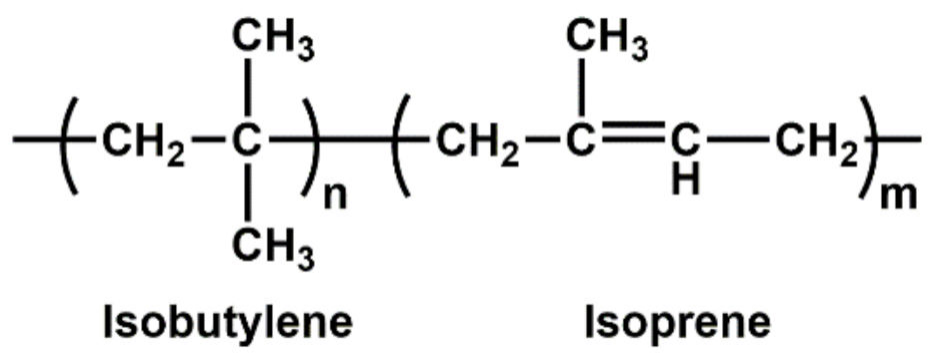
PU	Good abrasion resistance, tensile strength, tear strength, hardness, compression strength, impact resistance and elongation; high moisture sensitivity	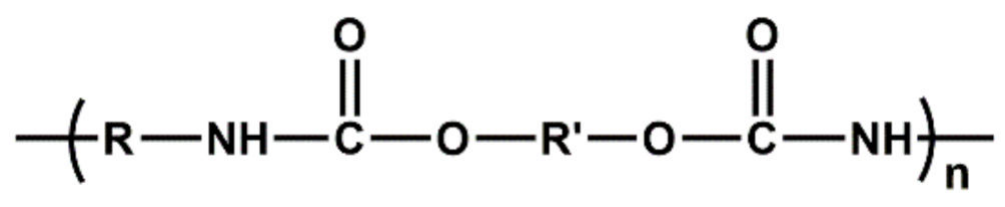
CPE	High resistance to ozone, hydrocarbon oil, heat and weathering; good blending ability with both polar and nonpolar materials	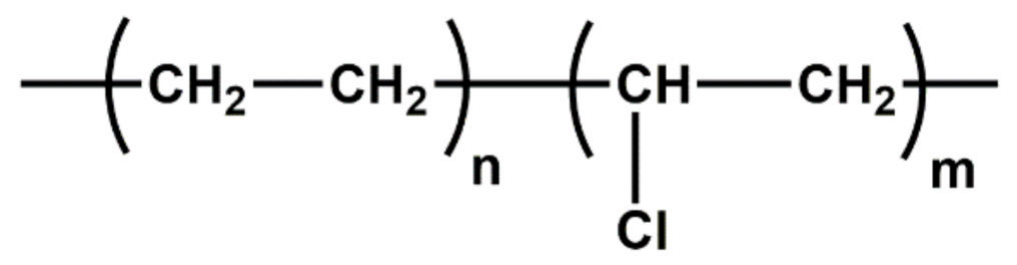
SR	Biocompatibility; high thermal and UV radiation stability; high elongation, elasticity and tensile modulus; good heat aging (100 °C) and compression set rating; adhesion to metals; good ozone, oxidation and weathering resistance; low resistance to alkaline solvents; good dielectric properties	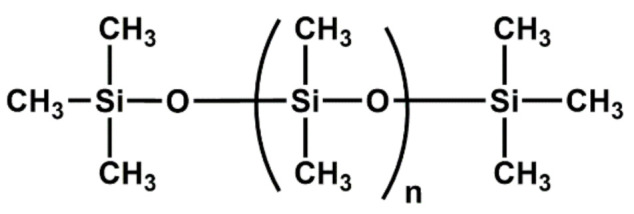

**Table 2:** Natural rubber grades with their impurities content [109].

Impurities	SMR-L	SMR-5	SMR-10	SMR-20
Dirt (max) wt.%	0.02	0.05–0.08	0.08	0.16
Ash (max) wt.%	0.50	0.60–0.75	0.75	1.00
Nitrogen (max) wt.%	0.60	0.60	0.60	0.60
Volatile matter (max) wt.%	0.50	0.80	0.80	0.80

**Table 3:** Principal groups of accelerators used in the sulfur vulcanization of elastomeric foams [151,153].

Group	Name	Properties	Structure
Amines	Diphenyl guanidine	Medium accelerator used along with other accelerators	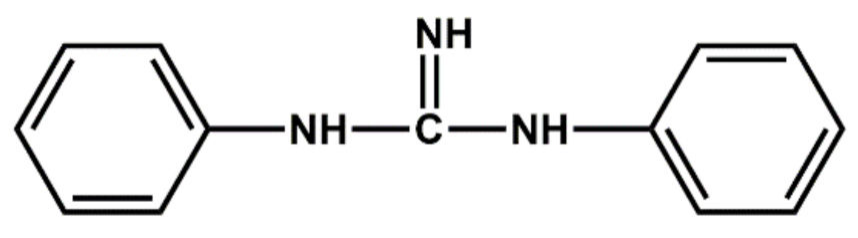
	Di-orthotolyl guanidine	Medium accelerator	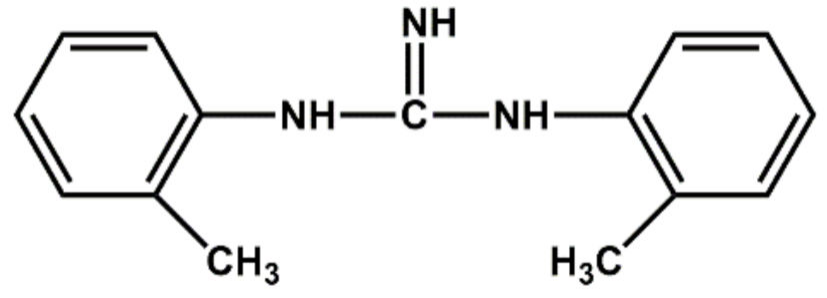
Dithiocarbamates	ZDEC	Ultra-accelerator	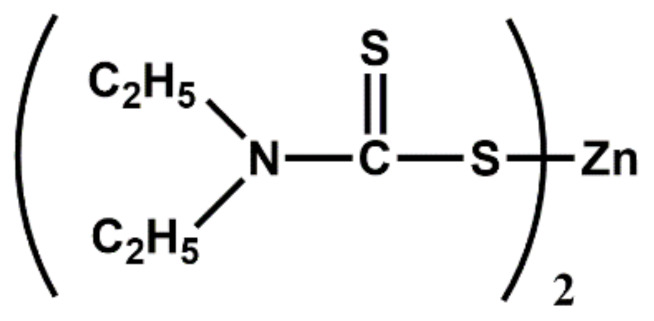
Xanthates	ZIX	Ultra-accelerator, low temperature curing	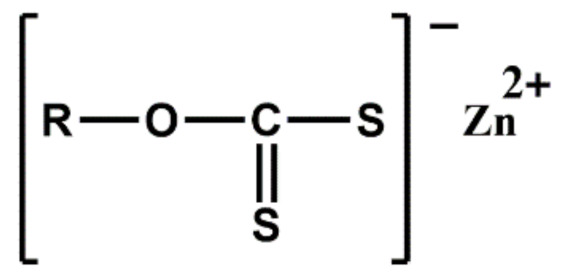
	SIX	Water soluble ultra-accelerator for latex work	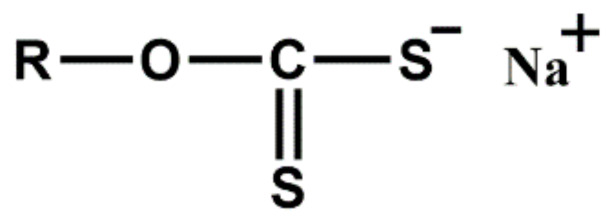
Benzothiazoles	MBT	Semi-ultra-accelerator, scorchy	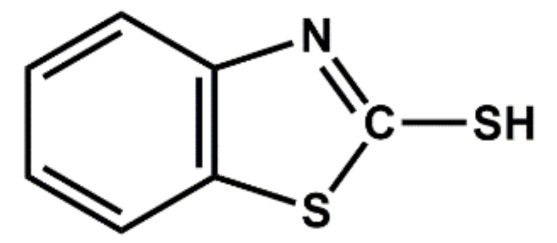
	MBTS	Delayed action, semi-ultra-accelerator	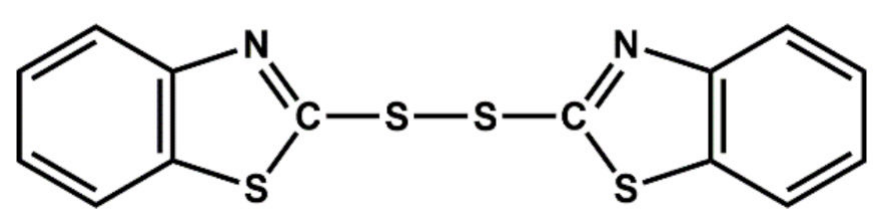
Sulphenamides	CBS	Delayed action, semi-ultra-accelerator	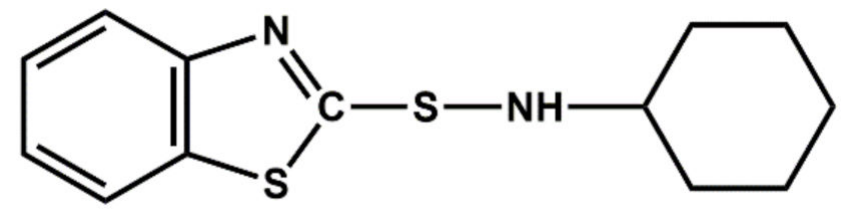
	TBBS	Delayed action	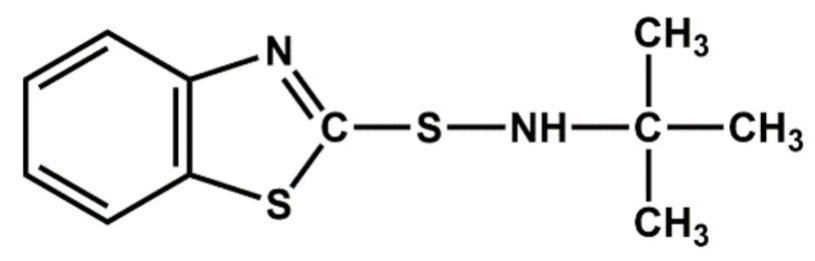
	MBS	Delayed action	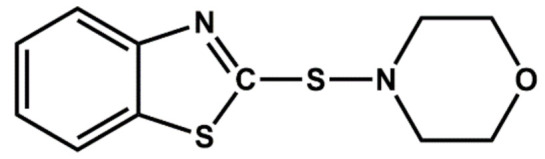
	DCBS	Delayed action, accelerator	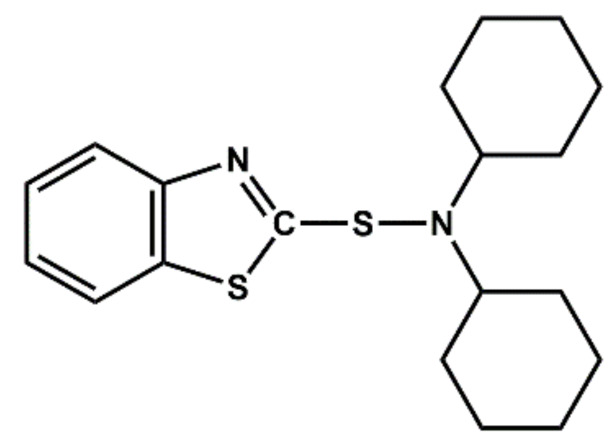
Thiurams	TMTM	Ultra-accelerator	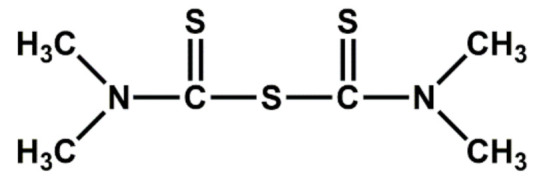
	TMTD	Ultra-accelerator, vulcanizing agent	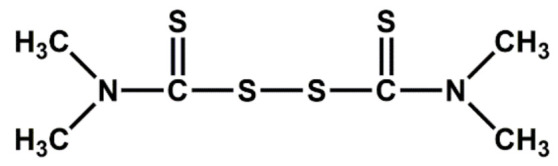

**Table 4:** Typical composition of rubber foam compounds [154].

Accelerators	NR (g)	ZnO (phr)	Steric Acid (phr)	Sulfur (phr)	ADC (phr)	Accelerators	
						Content (phr)	(Acc:S) ^1^ (mol)
MBS	40	5	2	0.5	4	2.5	5.08
TBBS	40	5	2	0.5	4	2.5	5.38
CBS	40	5	2	0.5	4	2.5	4.85
CBS	40	5	2	0.5	4	2.775	5.38

^1^ Ratio of accelerators to sulfur.

**Table 5:** Comparison between the main rubber foaming methods: batch, extrusion and injection molding.

Technique	Formation Method	Structure	Properties
Batch foaming	After masticating the neat rubber, all the ingredients, except for the curing agent, are added and mixing is performed for several minutes. Finally, the curing agent is added and mixed. The prepared compound is placed in a stainless-steel mold. Thereafter, the mold is put inside a high-pressure vessel (autoclave) at a defined pressure, temperature and time depending on the curing and foaming kinetics. After being saturated, the foamed parts are obtained by a rapid depressurization.	- Uniform cell size distribution can be achieved.	- Batch-wise process (time consuming). - Easy control for producing specific products. - Relatively low cost process compared to the other two techniques (no complex machine and equipment). - No shear: appropriate for producing sensitive materials. - High expansion ratio can be achieved.
Foam extrusion	The prepared rubber compounds in strip or pellet forms are fed into the extruder through the feed hopper and drawn into the rotating screw. The screw rotation continuously pushes the melt forward and out of the extruder through the die. The final part of this process is calibration and cutting of the extruded profiles.	- Achieving uniform cell size distribution is often difficult (faster cooling of the surface compared to the the middle of the sample).	- Continuous process (more difficult to control, but large scale production). - Thicker skinned foams. - The surface of the foams are often rough or/and have defects.
Foam injection molding	After adding the prepared rubber compounds into the screw in the shape of pellets through the hopper, the injection unit melts the rubber compounds and maintains the injection pressure during mold filling. Then, the gas-filled melt is injected through the nozzle into the mold at high temperature and pressure. Finally, the mold is opened, and a rapid pressure drop occurs leading to foaming.	- Achieving a foam with uniform cell sizes is difficult (the filling and maintaining time are coupled leading to difficult cell nucleation over the sample).	- Semi-continuous process (medium to large scale foam production). - Produce samples with a wide range of geometry and size. - Low material use. - High dimensional stability of the parts. - Low back pressure required. - Wide range of mechanical properties with very high stiffness-to-weight ratio.

## Data Availability

Not applicable.
